# Influence of Magnitude and Duration of Altered Gravity and Readaptation to 1 g on the Structure and Function of the Utricle in Toadfish, *Opsanus tau*

**DOI:** 10.3389/fphys.2018.01469

**Published:** 2018-10-22

**Authors:** Richard Boyle, Yekaterina Popova, Joseph Varelas

**Affiliations:** ^1^National Aeronautics and Space Administration Ames Research Center, Moffett Field, CA, United States; ^2^Universities Space Research Association (USRA) Science & Technology Innovation Labs at NASA Ames Research Center, Moffett Field, CA, United States

**Keywords:** afferents, hair cells, acceleration, gravity, centrifugation, spaceflight, electrophysiology, serial electron microscopy reconstruction

## Abstract

Gravity has remained constant during animal evolution and the neural sensory systems detecting acceleration forces have remained remarkably conserved among vertebrates. The utricular organ senses the sum of inertial force due to head translation and head tilt relative to gravitational vertical. Change in gravitational force would be expected to have profound effects on how an organism maintains equilibrium. We characterize the physiology of utricular afferents to applied accelerations in the oyster toadfish, *Opsanus tau*, in normal 1 g to establish benchmarks, after 1–32-day exposures to 2.24 g (resultant) via centrifugation (hypergravity, HG), after 4- and 16-day exposures to 1.12 g (resultant), and following 1–8 days recovery to HG exposures to study re-adaptation to 1 g. Afferents were also examined during activation of efferent vestibular pathway. Centrifugation at 2.24 g included 228°/s constant angular velocity component, and thus horizontal canal afferent responses to yaw rotation were recorded as an internal control in each fish. Afferents studied after 228°/s rotation for 4 and 16 days without centripetal acceleration, called On-Center-Control, were indistinguishable from their control counterparts. Principal response to HG was an adjustment of afferent sensitivity as a function of magnitude and duration of exposure: an initial robust increase at 3–4 days followed by a significant decrease from 16 to 32 days. Initial increase observed after 4 days of HG took >4 days in 1 g to recover, and the decrease observed after 16 days of HG took >2 days to readapt to 1 g. Hair cells in striola and medial extrastriola macula regions were serially reconstructed in 3D from thin sections using transmission electron microscopy in control fish and fish exposed to 4 and 16 days of HG. Despite the highly significant differences in afferent physiology, synaptic body counts quantified in the *same* fish were equivalent in their inter-animal variability and averages. No clear role of the efferent pathway as a feedback mechanism regulating afferent behavior to HG was found. Transfer from 1 g to HG imparts profound effects on gravitational sensitivity of utricular afferents and the accompanying transfer from the HG back to the 1 g resembles in part (as an analog) the transfer from 1 g to the micrograms.

## Introduction

Gravity is a natural force that drives all living organisms to acquire precise physiological mechanisms to survive. Throughout the course of a species’ evolution the intensity and direction of gravity have remained constant, whereas other environmental factors, such as predation, climate, vegetation, for example, with rare exceptions have not. The highly evolved inner ear neural systems in vertebrates are keyed to operating under gravity’s continuous influence (Stensiö, 1927; Friedman and Giles, 2017). Detections of inertial acceleration, such as linear translation during self-motion or an external perturbation, and head orientation with respect to gravity, such as tilts, start from the bilateral otolith organs. The first vertebrate ear was principally a graviceptive statocyst more commonly found in today’s aquatic invertebrates (Budelmann, 1988). Otolith structures contain an inertial mass of calcium carbonate that is subject to the pull of gravity and thus possesses weight. In an earlier study (Boyle et al., 2001) fish were exposed to near weightlessness in spaceflight and in this study we exposed the fish to centrifugation to enhance the gravity load and queried in a similar fashion the sensory processing characteristics of otolith afferents.

Pervasiveness of gravity provides the nervous system a common reference about which to optimize sensory transduction mechanisms and perception. Otolith afferents carry the transduced signal of the gravito-inertial forces in the form of modulated spacing of action potentials to the brainstem and cerebellum (see Goldberg et al., 2012). There the otolith code combines with angular acceleration signals obtained from the semicircular canals and with information derived from the visual and somesthetic modalities to compute a central representation of the body and its parts as well as their dynamics in space. The end result is the resolution of ambiguity of gravity and self-motion to allow healthy individuals to maintain balance and equilibrium under varying conditions. Despite invariability of gravity on Earth, physiological systems are not and are adversely affected by trauma, disease, and aging, and thus central creation of the gravito-inertial vector can be disrupted leading to vertigo, disorientation, and other clinical disorders.

Arrival of aviation and space age offered a means to directly study the role of gravity in healthy subjects. The established reference of self-orientation is now completely violated by the speed at which the individual and visual world on the retina move and by rapidly changing orientation of the gravity vector with respect to the pilot, or removal of gravity vector altogether in space missions. During the first days of spaceflight, most astronauts experience symptoms of space motion sickness and disorientation (Reschke et al., 1994; Demontis et al., 2017) linked to the otolith organs (Watt et al., 1986; Paloski et al., 1993; Heer and Paloski, 2006). An extraordinary hypersensitivity to applied acceleration was observed in toadfish utricle afferents after relatively short mission aboard Shuttle orbiters (Boyle et al., 2001). The hypersensitivity presumably was the result of exposure to near weightlessness, persisted over the first day after landing, returned to within normal levels on the second day at 1 g, and closely matched the reported readaptation in astronauts after landing following a comparable stay in space (Reschke et al., 1994).

Physiological adjustments to a novel gravity, as witnessed in spaceflight, reveal that the organism possesses adaptive mechanisms triggered by transitions in gravity. What initiates these mechanisms and are they compensatory to the gravity load? In addition to the sensory afferent pathways the brain in turn sends efferent signals back to the same inner ear structures. Activation of olivo-cochlear efferent neurons can attenuate afferent sensitivity and reduce its frequency selectivity or tuning to sound (Warr and Guinan, 1979; Liberman, 1980; Groff and Liberman, 2003). In functional terms, this efferent-to-afferent feedback loop adjusts sensitivity of hearing in a frequency dependent way (Brown et al., 1983; Russell and Murugasu, 1997). Analogous to the cochlea, efferent synaptic terminals contact both vestibular hair cells and afferents (Smith and Rasmussen, 1968; Sans and Highstein, 1984; Holstein et al., 2004; Lysakowski and Goldberg, 2008), and activation of efferent synapses influences afferent sensitivity in fish (Flock and Russell, 1976; Hartmann and Klinke, 1980; Boyle and Highstein, 1990b; Boyle et al., 2009), frog (Gribenski and Caston, 1976; Valli et al., 1986; Chagnaud et al., 2015), turtle (Brichta and Goldberg, 2000; Holt et al., 2006), bird (Dickman and Correia, 1992), chinchilla (Marlinski et al., 2004), and primates (Goldberg and Fernández, 1980; Sadeghi et al., 2009). Reflex or self-generated movements often have large purposeful acceleration components, such as those occurring during predation (attack) and avoidance (escape) behaviors that would impair the computation of a movement, and importantly any novel perturbations encountered during execution of those behaviors. To enable the afferent to continuously encode motion profiles during such overt behaviors its sensitivity to acceleration is reduced, often dramatically, but it is not clear whether the prevailing gravity load is registered by the efferent system and participates in adaptive processes.

Another possible participant in adaptive processes in the peripheral labyrinth is a rapid addition or removal of synaptic bodies associated with the hair cell-afferent synapse. Ross (2000) found the number of synaptic ribbons in rat type II otolith hair cells increased two to threefold following 14-day exposure to weightlessness. Since toadfish possess only type II hair cells, an attractive interpretation of our earlier afferent data (Boyle et al., 2001) was an increase in synaptic strength as an initial adaptive response to restore the “lost” gravity detection, followed by a deletion of the added synaptic bodies (Waites et al., 2005; Matthews and Fuchs, 2010; Graydon et al., 2017) leading to a restoration of normal sensation after a return to 1 g. Here, we manipulate the afferent responses to acceleration by applying variable exposures to hypergravity (HG) via centrifugation and directly correlate the observed afferent sensitivity with serial reconstruction of hair cell-afferent complexes in two areas of the utricular macula in the *same* animal.

## Materials and Methods

### General Procedures

Oyster toadfish, *Opsanus tau*, of either sex, weighing ∼400 g, were provided by the Resources Facilities of Marine Biological Laboratory (Woods Hole, MA, United States). Fish were housed in 300 l saltwater aquaria systems (AquaLogic, San Diego, CA, United States) and monitored daily by the veterinarian staff. All procedures followed the principles set forth by NIH “Guide for the Care and Use of Laboratory Animals, Ed 8” and approved by NASA Ames Research Center Institutional Animal Care and Use Committee. Useful data were acquired from 106 fish.

Immediately after removal from the centrifuge aquarium (<1 min) the fish was immersed in the anesthetic agent MS222 (25 mg/l, 3-aminobenzoic acid ethyl ester, Sigma, St. Louis, MO, United States) diluted in saltwater (Instant Ocean^®^). Fish was then placed in a Lexan polycarbonate tank filled with chilled (15°C) saltwater, and its head was stabilized in an Earth horizontal plane using a mouth brace and immobilized with stainless steel pins to the cranium; saltwater covered all but its most dorsal surface, and its eyes and dorsal fins were covered with moist tissues. Saltwater was aerated using an air stone. MS222 was added as needed during the course of the experiment.

A small craniotomy was made to visualize the anterior and horizontal canal (HC) ampullae and the utricle and their specific nerve supply. Fluorocarbon (FC75, 3M Corp, Minneapolis, MN, United States) was injected into the cranial opening to fill the perilymphatic vestibule. The dorsal surfaces of the brainstem and posterior cerebellum were exposed by removing ∼3 mm^2^ portion of occiput above the foramen magnum. Efferent vestibular nuclei are seen as paramedial gray areas among the transversely running internal arcuate fibers under the floor of the IVth ventricle (Highstein and Baker, 1986). A pair of sharpened Ag/AgCl wires insulated to within 0.2 mm of their tips and spaced ∼2 mm was lowered into the efferent nuclei on either side of the midline along a ∼45° angle beneath the posterior cerebellum to a depth of 1–2 mm. EVS activation behaviorally provoked or electrically induced elicits an early arousal reaction, characterized by an initial flaring of lateral fins and raising of dorsal fin, followed by straightening of the tail and extension of the ventral fins (Highstein and Baker, 1985; Boyle and Highstein, 1990b). Fin flaring was used as a guide for proper placement of electrode array that required the lowest shock intensity. Alert fish activate their EVS at a maximum rate of ∼100 impulses/s (ips) (Highstein and Baker, 1985), and efferent nerves faithfully follow a 100 Hz stimulus train applied to the nuclei (Boyle and Highstein, 1990b). EVS stimulation consisted of single pulses of 100 μs duration with trains of 100/s were delivered in a bipolar fashion via a pulse generator coupled to a stimulus isolation unit and captured as time-stamped events in Spike2 (CED, United Kingdom). Conventional 30–50 MΩ glass microelectrodes, filled with 2 M LiCl_2_, were used for extracellular or pseudo-intra-axonal recordings under direct visual control. The recording session began less than 1 h after cessation of centrifugation and typically lasted 12–14 h.

In preliminary experiments, we noticed utricular afferents were inconsistently responsive to EVS stimulation, while at the same time HC afferents were consistently activated. Therefore, responses of ∼5–10 HC afferents were recorded at the start of each session to adjust and confirm efferent stimulation parameters. Sinusoids of 10–20°/s at 0.5–5 Hz were delivered about the *z*-axis (yaw rotation) as well as visual localization to identify canal afferents. Consistency of stimulus intensity was verified throughout the experiment. Another set of canal afferents (range 8–27) was recorded at the end of each session to confirm EVS efficacy and evaluate canal response to rotation.

### Centrifugation Procedures

**FIGURE 1:**
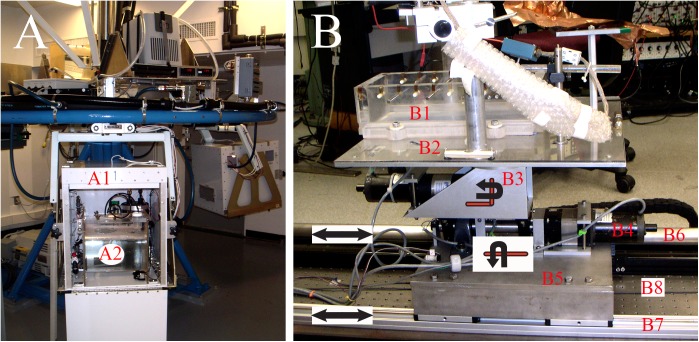
Experimental and testing devices. **(A)** Ground-based centrifuge was used to apply centripetal force by constant velocity rotation within a concomitant 1 g environment. Centrifuge had four gondolas (labeled A1) positioned at a radial distance of 1.22 m from center of axis of rotation. Each gondola was gimbaled with one-degree of freedom to deliver a constant gravity vector onto fish at any given rotational rate. Self-contained aquarium systems (labeled A2) were developed to permit life support for one fish in each gondola consisting of mechanical and biological filtrations, cooling, and oxygenation within a 12:12 light:dark cycle. Fish were unrestrained during centrifugation and their behavior was captured in light using video. **(B)** Acceleration testing device was designed and fabricated to study afferent responses to controlled translational motions delivered along the tandem rails labeled B6 and B7 at any head orientation via yaw rotation controlled by motor labeled B3. The motor labeled B4 delivered tilt stimulation from pitch to roll. See text for more details.

NASA Ames prototype centrifuge was used to subject the fish to HG of variable duration and magnitude (Figure 1A). The centrifuge consists of four radial arms with a one-degree of freedom swing gondola (labeled A1) at the end of each arm to maintain gravity vector relative to the fish during rest and centrifugation and positioned 1.22 m eccentric to center of rotation. Two magnitudes of HG were applied. Main protocol was a 38-rpm spin delivering a 1 g (Earth) plus 2 G (centripetal) force imparting a 2.24 g (=sqrt(1^2^ + 2^2^)) resultant force. Duration of 2.24 g exposures was 1, 2, 3, 4, 5, 8, 16, 24, or 32 days in groups of four to five fish. In separate experiments, the spin was reduced to 19 rpm resulting in a 1 g centripetal force or 1.12 g (=sqrt(1^2^ + 1^2^)) resultant force; duration of 1.12 g exposures in groups of four fish was confined to 4 and 16 days. Loading and unloading of fish were scheduled to minimize the stop time, typically <10 min. Twelve separate runs were conducted over a 3-year period to acquire the reported HG results.

Fully sealed fish tanks were fabricated from 1.3-cm Plexiglas^®^ tubes of 30.5-cm dia capped at both ends, and with a molded, sealable opening on the curved surface to permit access to the fish. An individual habitat (labeled A2) was placed in each gondola, contained ∼20 l of saltwater and housed either one fish or none at a time (for scheduling). A closed loop system continuously circulated water through mechanical and biological filtrations and a heat exchanger to chill the saltwater to 15°C on the return side. The room was cooled to <15°C to counter heat generated by the motor. A DC powered air stone aerated the saltwater, and pressure was released via a one-way valve. Light/dark (12:12 h) cycles during centrifugation were normalized to home aquaria. Fish were unrestrained and allowed to swim freely in the habitat. During centrifugation, two forces are applied to the fish in addition to the constant force of gravity: a centripetal force dependent on radius of eccentricity and rate of rotation. To account for possible variations in vestibular function due to rate of rotation, a separate rate table was configured that permitted a single tank to be placed precisely at the center of rotation axis. These On-Center Control (OCC) experiments were conducted one fish at a time with a spin of 38 rpm for groups of four fish at either 4- or 16-day duration. All parameters were matched to the centrifuged fish with the exception that no (or negligible) centripetal force was imparted to the fish. Fish were monitored in the light cycle using video. No off-nominal events occurred during any test.

#### Acceleration Testing Device

After surgical preparation the tank (labeled B1) was mounted to a multi-axis linear and angular acceleration and tilt system (Figure 1B). Head position of the fish was adjusted to align the otoliths coplanar to Earth horizon using digital commands to one or more axes. The cranial opening was positioned beneath a castle-like structure on which a three-axes remotely controlled motorized micromanipulator (MP-285; Sutter Instruments, Novato, CA, United States) and preamplifier were fixed.

The fish tank and castle-like platform were attached to a Plexiglas^®^ plate (labeled B2) supported by a 15.24-cm dia thrust bearing and attached to a stepper motor (labeled B3) geared (55:1) at 90° (graphically illustrated) to provide a full rotation about the vertical *z*-axis (yaw); one stepper count equals 0.00026°. This axis allows smooth positioning from one head angle to another with respect to the translational axis to examine afferent directional selectivity, together with applied yaw rotational stimuli. Its center of rotation passed through the center of the head and bilateral labyrinths. Yaw stage was mounted to a 2.54-cm dia shaft through roller bearing pillow blocks to a stepper motor (labeled B4) straight geared (55:1); one stepper count equals 0.00026°. This axis allowed tilt about the axis coplanar to the Earth horizontal (graphically illustrated), and provided either static positioning or dynamic stimuli at angles between pure roll and pure pitch depending on the head angle command to the yaw motor. Next, the tilt stage was attached to a base plate (labeled B5) through roll bearing pillow blocks and stepper motor mount. The base plate was attached to a thrust block brushless linear motor (not visible in Figure 1B), which was in turn mounted to a 165-cm precision linear bearing guidance system (IDC, Petaluma, CA, United States) (labeled B6); a non-contact, incremental linear encoder provided a position sensing with 10 μm accuracy and voltage proportional to instantaneous linear velocity was derived from the servo controller; this signal was integrated in Spike2 data acquisition software to obtain a derived acceleration signal. A second, slaved rail, and linear bearing gliders (labeled B7) were coupled to the thrust block to provide additional stability and lower the center of mass of the top plate. The device was secured to an optical breadboard (TMC) (labeled B8) to ensure flatness and provide isolation, and floated from the lab floor by jelly pads to absorb substrate-borne vibrations. Unit recording stability was confirmed up to 0.5 g at 12 Hz (maximum tested). A digital controller (Galil Motion Control, Rocklin, CA, United States) installed on a Windows-based platform provided command signals to each motor. Software control was based on Galil motion control libraries called from a user interface written in Igor (WaveMetrics, Lake Oswego, OR, United States) scripts to permit step or continuous, dynamic motion about one or more axes at selectable parameters.

### Terminology of Motion Stimuli

*Lab-defined frame of motion* reference along the horizontal axis at 0° corresponded to a positive acceleration directed forward out the fish’s snout; activation of the tilt stage (Figure 1B, B4) at this position delivered a *pure roll* to one side or the other. Activation of the yaw stage (Figure 1B, B3) produced counter-clockwise (CCW) or CW rotation in the horizontal plane to change the head orientation with respect to the linear motion. Yaw rotation of the platform to + 90° (right-hand rule, CCW) about the *z*-axis produced a positive inter-aural acceleration directed out the right (ipsilateral to recording side) ear and the converse out the opposite ear, and activation of the tilt stage produced a *pure pitch* with either head-down or -up. Yaw rotation of the platform to 180° about the *z*-axis produced a positive sled acceleration directed forward but now out the fish’s tail, and activation of the tilt stage produced again a *pure roll* to one side or the other. Yaw rotation of platform to 270° about the *z*-axis produced a positive acceleration directed out the left (contralateral to recording side) ear and the converse out the right ear, and activation of the tilt stage produced again a *pure pitch*. Sinusoidal translation along the horizontal plane ranged from 0.03 to 0.3 g at 0.3–5 Hz and was limited by the excursion of the rail; sinusoidal tilt stimuli ranged from 0.01 to 2° at 0.3–10 Hz and up to 5° static tilt and the parameters tested were dependent mainly on recording stability; sinusoidal yaw rotations were confined to 10–30°/s at 0.1–5 Hz.

### Vestibular Test Protocol

Test protocol was designed to provide direct comparisons of afferent responses to translation within and across the populations subjected to different HG and adaptation conditions. Secondarily, tilt stimuli were used to extend the response characteristics. The hair cell’s bundle is morphologically polarized (Wersäll, 1972) and its receptor potential is directionally sensitive and behaves as a rectified cosine-function to bundle displacement (Hudspeth and Corey, 1977; Shotwell et al., 1981); the functional polarization vectors of utricular afferents are distributed in a fanlike shape (Fernández and Goldberg, 1976b), as is to be expected from the hair cell orientations in the macula (Spoendlin, 1964). Afferent directional selectivity was defined by its maximum (Smax) and minimum or null (Smin) sensitivities. To accomplish this a sinusoidal translation along a plane parallel to Earth was delivered as the fish was rotated about the yaw or *z*-axis (Angelaki and Dickman, 2000; Boyle et al., 2001) and paused to collect five or more consecutive cycles. In our standard test protocol, a 1–2 Hz translational sinusoid was delivered at amplitude of ±0.02–0.05 g as the fish was stepped about the *z*-axis in typically successive 15° steps. Test magnitude was set to prevent *as well as possible* any nonlinear distortions of afferent impulse rate (IR) such as saturation in the ON-direction or “cut off” in the OFF-direction. In successful recordings, the afferent Smax and Smin to translation were captured within five to nine steps; in some cases, the Smin was not obvious (discussed later in the section “Results”). In many cases the recording was compromised before completing the required protocol and the data were discarded. In afferents with particularly stable recording conditions static and dynamic tilts were delivered, selected often at the *z*-axis angle that elicited its Smin and Smax responses to translations. Static tilts were typically ±1–3° displacement (12°/s^2^ ramp on/off) and were held to determine the time course of impulse adaptation. In some instances afferent directional selectivity was determined for both translations and tilts.

### Data Acquisition and Analysis

Data waveforms and events were digitized, displayed in real-time using Spike2 software, and stored for later analysis (CED Power 1401). Direct voltage measures of angular displacements (in degree) about the two (yaw and tilt) rotational axes, acceleration (cm/s^2^) from the linear encoder and the derived velocity (cm/s), and the linear accelerators (g) along the horizontal plane were amplified externally to span the full 16-bit range of the A/D converters, filtered below the Nyquist frequency, and digitized at 500–1000 samples/s. Time stamping for stimulus trigger events, efferent shock times, and externally discriminated spike times was acquired to a resolution of 0.08 ms.

Data were imported off-line into a custom interactive analysis procedure written in Igor. Desired portions of record with respect to specific stimuli were manually selected and inspected using an interactive graphical analysis procedure. A stimulus trigger (positive zero-crossing) was used as a time reference to generate 100–180 bin/cycle phase histograms of stimulus and response. A discrete Fourier analysis was applied to compute the first-harmonic stimulus/response amplitude and phase. Response sensitivity (afferent output/stimulus input) was calculated by dividing IR modulation of derived 1st harmonic of modulation by acceleration amplitude, e.g., impulse rate (IR) per second (ips) per cm/s^2^ or g (9.81 m/s^2^) linear acceleration; response phase is angle between output and input peaks in ° using the lab-defined reference. A cosine function was applied to data in a method following (Angelaki, 1991; Angelaki et al., 1993) to determine degree of spatial tuning, directional selectivity, and response maximum (Smax) and its minimum (Smin). Some afferents are silenced over a portion of the stimulus cycle. In the least squares fitting procedure bins lacking data were not assigned a value of zero, and therefore the curve fit was not influenced by a truncated response. This form of response nonlinearity may lead to a misrepresentation of data because a portion of the curve fit will take negative values. When detected in real time, the stimulus was reduced in amplitude to permit a more full-cycle impulse modulation. Despite these efforts and the need to maintain comparable stimulus parameters for capturing Smax response saturation and silencing of the discharge were often unavoidable. Both the mean IR, defined as IR at half of computed peak-to-peak modulation, and the average IR, defined as number of impulses divided by stimulus cycle time, were measured. The former IR assists in detecting large truncated or clipped responses and the latter took into account the different cycle time of sinusoids used and served as a more reliable measure of IR during stimulation. Samples of IR >30 s in length (from 120 to >36,000 interspike intervals) in the absence of stimulation were taken to describe the afferent background impulse properties. Degree of regularity was evaluated by determining the non-normalized coefficient of variation (CV) of rate, defined as the standard deviation of interspike intervals divided by mean interval. An example of protocol for characterizing afferent responses in control and experimental fish to linear acceleration (panels A–C) and to tilt (panel D) is given in Figure 3.

Statistical afferent data analyses were performed using built-in one-way ANOVA packages in Igor (v.7) and KleidaGraph (v. 4.5) with *post hoc* analysis (Tukey’s all pairs comparison test). Data are presented throughout as the mean ± SD as single group and compared as paired data using Student’s *t*-test and levels of statistical probability are two-tailed values. Differences were considered significant for *p* < 0.05.

### Histology

At the end of each experiment a small opening was made in the dorsal aspect of the anterior canal limb, and a glass pipette with ∼50 μm opening was inserted into canal lumen and directed rostrally toward the anterior ampulla. The pipette was attached to a 1 ml syringe, and the endolymph was flushed with fixative solution containing 2% paraformaldehyde, 2.5% glutaraldehyde, 0.1 M sucrose, and 0.2% picric acid in 0.1 M phosphate buffer (pH 7.4). Perfusate was colorized using alcian blue to visualize it flowing across the anterior ampulla, entering the utricular space and duct, and exiting via the common crus from the distal end of the cut canal limb. The utricle and a portion of its nerve were extracted, and the utricle was opened to expose the macula and immersed in fixative overnight. Maculae were post-fixed in 2% osmium tetroxide with 1.25% potassium ferrocyanide, dehydrated, infiltrated, and embedded in Epon. Blocks were sectioned using a Leica Ultracut UCT ultramicrotome in 1-μm thick sections for surveying and 180 nm sections for analysis.

### Synaptic Body Counts

To provide uniformity of synaptic body (SB) counts within our samples and allow direct comparison with a previous work, a protocol based on Ross (2000) was used. The Epon block was cut in 1 μm sections along the short axis of macula to survey the start of the striola border. Once confirmed 100 sections at 1 μm thickness were collected for a separate morphometric evaluation. From that point 150 serial sections of 180 nm thickness were collected to characterize the striola region on both sides of the hair bundle reversal line; a separate comparable collection of serial thin sections was obtained 150 μm medially to characterize the medial extrastriola zone of the macula. Sections were collected individually, mounted on Butvar-coated slot grids, and studied with a Leo 912 AB transmission electron microscope. More details on the ultrastructure of the toadfish utricle and the three-dimensional reconstruction of hair cells can be found in Boyle et al. (2018).

**FIGURE 2:**
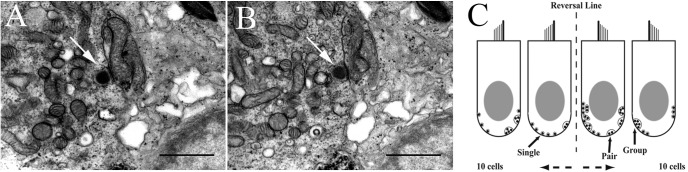
Method to identify and count synaptic bodies (ribbons) in utricular hair cells. **(A, B)** Two consecutive transmission electron microscopic images (12,500× magnification) reveal the same SB (arrow in each panel) in the hair cell. Scale bar, 1 μm. **(C)** Two-dimensional cartoon of method used to tabulate and characterize the unique synaptic bodies in each hair cell reconstructed in 3D from 150 serial sections of 180 nm thickness. Two 100 μm width patches separated by 150 μm in striola (shown with the reversal line) and medial extrastriola regions of macula were examined in same fish in which afferent recordings were collected. Sections were viewed at 500–20,000× to verify synaptic bodies within the section and carried over into adjacent sections. Synapse characterization is based on following criteria. Single body: most common and do not share synaptic vesicles with nearby bodies if present. Pair: less common with two synaptic bodies sharing vesicles on one section, or adjacent sections. Group: least common with ≥3 bodies sharing vesicles on one section, or across multiple serial sections.

Each sample was coded to prevent detection of treatment (control or HG) and experiment day. Digital mosaics were made using the Olympus Soft Imaging System MegaView III digital camera at 5000× (microscopic) magnification from every seventh section and the hair cells were numbered. Mosaicked sections served as maps for locating and identifying the numbered hair cells in non-mosaic sections. Every hair cell on every section (mosaic and non-mosaic) was examined for presence of synaptic bodies and hair cell-afferent synapses. Sections were initially analyzed at 8000× magnification, and suspected synapses were enlarged to 12,500× for validation (see Figures 2A,B). Digital images of all likely synaptic bodies were taken and coded with a number for the section, hair cell, and number of synapses present. A low power image (4000×) was also taken for better cell identification on the map. Combined data were used to accept or reject probable synaptic bodies. Synapses or other structural features of interest were also photographed at this time. Images were transferred to an SGI Onyx for permanent storage and further synapse analysis. For counting, synapses were recorded on sheets of paper on which there are numbered contours of identified hair cells, with the numbers matching those on the maps. Strict criteria for counting synaptic bodies were followed. Only structures consisting of a central, electron-opaque body (presynaptic density), and a halo of vesicles were counted. Panels A and B in Figure 2 show digital images of two consecutive sections separated by section thickness of 180 nm. Arrows in each panel point to the same single SB in the hair cell. Figure 2C shows a cartoon of 2 of the 10 hair cells on either side of the reversal line in the striola as an illustration of method used in tabulating the results. Greater than 90% of synaptic bodies are single specializations along the hair cell internal wall, followed by pairs of bodies, and rarely a cluster of three or more bodies. Because of the time required to perform this task, typically 3–4 months full-time effort per macula, SB counts were done from selected fish in which extensive physiological data were first obtained. Synaptic ribbons in utricular hair cells were analyzed using the ANOVA and MANOVA features of SuperANOVA^TM^ software. The level of significance was set at *p* < 0.05.

## Results

### Control Responses to Translational Accelerations and Tilts in *Normal Gravity*

**FIGURE 3:**
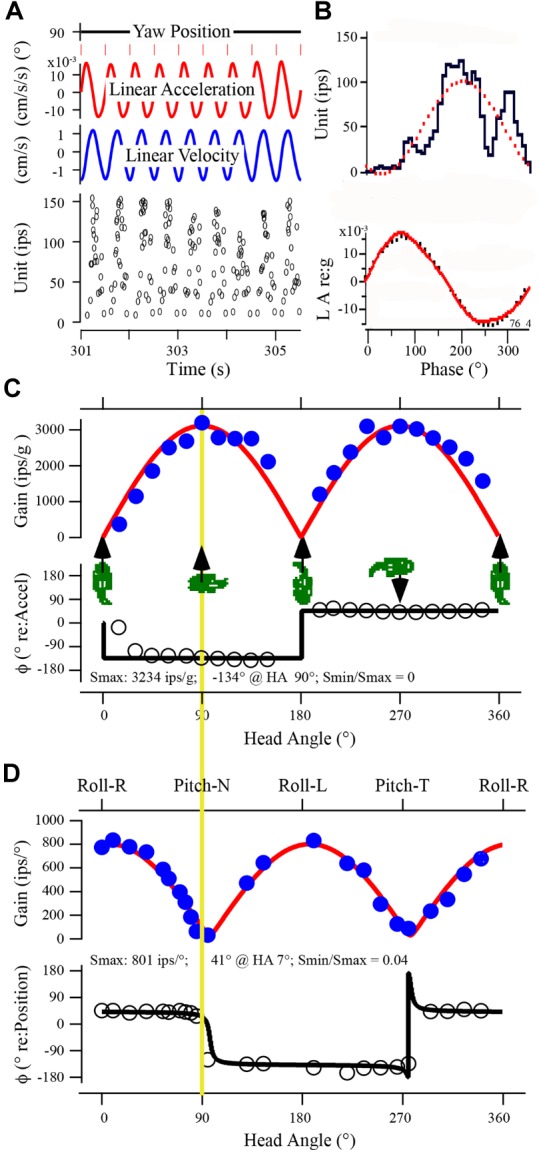
Uricular afferent response properties. **(A)** Recorded data of afferent response in control fish show IR modulation to sinusoidal linear acceleration at 2 Hz collected over time from 301 to 305.5 s. Traces from top to bottom: (1) yaw position of fish (set at 90°). (2) Linear acceleration of sled in ±cm/s^2^. (3) Linear velocity of sled in ±cm/s. (4) Instantaneous IR in impulses per second (ips). **(B)** Data in **A** were averaged and displayed as phase histogram; upper trace is unit IR (ips) and lower trace is stimulus acceleration (LA) in g (dotted curves represent the 1st harmonic fit to the stimulus and response). **(C)** Directional selectivity of utricular afferents. Responses were analyzed at 21 separate orientations of fish in space shown in the abscissa from 0° (positive linear acceleration directed in horizontal plane out fish’s snout) to accelerations directed in horizontal plane out fish’s right (90°) or left (270°) ear and at orientations in between. Cartoons give at five positions of fish to help visualize the stimulus. Maximum response (in ips/g) was calculated at each orientation and plotted as a function of head angle (in °). A cosine function (red curve) was applied to data to determine degree of spatial tuning, directional selectivity, and response maximum (Smax) and its minimum (Smin). In this example Smax was 3234 ips/g at 90° (comparable at 270°) and afferent was highly spatially tuned (Smin/Smax = 0). **(D)** Responses of same afferent to sinusoidal tilt simulation at 2 Hz, ±0.11° displacement. Format is same as in **C**. Upper trace gives a broad description of stimulus, from Roll with Right ear down at 0°, Pitch with Nose down at 90°, Roll with Left ear down at 180°, and Pitch with Tail down at 270°. Yellow vertical line between **C** and **D** is given to provide the 90° offset of the two stimuli by the lab-defined coordinate scheme, and how closely the responses match. Smax for tilt was 801 ips/° at 7° head angle, and the spatial tuning was tight (Smin/Smax = 0.04).

Response properties of utricular afferent were studied in control fish housed in 1 g to establish baseline measures needed for direct comparison within and across fish exposed to the different experimental conditions. A representative utricular afferent behavior in control fish to linear acceleration (panels A–C) and to tilt (panel D) is given in Figure 3. Orientation of the fish about the *z*-axis (Yaw Position in A) was set at 90° to provide a positive acceleration directed out the fish’s right ear along the inter-aural axis ipsilateral to the recording site. Nine consecutive cycles of sinusoidal linear acceleration (±15.7 cm/s^2^ or ±0.016 g) at 2 Hz were recorded (A) and averaged (B) to give a response of the following characteristics: averaged modulation was ±51.7 ips about a fitted rate of 47 ips, with a sensitivity of 3.3 ips/cm/s^2^ or 3234 ips/g occurring at peak phase of -134° re: acceleration. Average IR was 41 ips/cycle indicating a slight silencing of its rate in the OFF-direction. This response represented the afferent Smax. Afferent directional selectivity, a critical parameter to measure across experimental fish to precisely establish Smax, the spatio-temporal characteristics of averaged data at different head angles are plotted in Figure 3C. Cartoons of fish show the direction of +linear acceleration at five different fish orientations. Smax (Figure 3C) is the uppermost point (solid circle) on the yellow line. Rotation of fish about the *z*-axis from Smax at 90° head angle (Figures 3A,B) by half-circle to 270° head angle elicited a comparable and expected Smax along the inter-aural axis but with a 180° phase shift due to the lab-specific denotation. Peak of response modulation with respect to peak acceleration amplitude typically remained consistent over a hemi-field of head angles, rectified as the polarity shifted in the lab-defined coordinates, and showed variable leads to lags. Data were fit by a rectified (temporal or phase of response; black curve superposed on open circle symbols of phase re: acceleration) cosine (amplitude of modulation) function (red curve in Figure 3C superposed on solid blue circle symbols of gain data). Note the rise and fall of gain in a cosine fashion as the fish’s orientation was shifted from inter-aural accelerations to accelerations directed out the fish’s snout or tail. The afferent was tested for nearly 360° change in orientation at 21 separate head angles. The fitted nulled value of 0 for Smin (e.g., Smin/Smax = 0 in the example in Figure 3C) reflected the sharpness of directional tuning and the correspondence between tested (filled and open circles) and fitted (red and black curve) responses was sharp. Same afferent’s responses to tilt stimuli at 2 Hz, ±0.16° displacement, were recorded in a similar fashion at 23 separate orientations to determine Smax and Smin to tilt (D). Smax was 801 ips/° and nearly aligned to pure *roll* tilt as expected from the inter-aural responses to linear acceleration. Smin was near a complete null (32 ips/°) response predicted from the Smax to linear acceleration (yellow line) at both nose down (Pitch-N) and tail down (Pitch-T) pitch tilts, and the Smin/Smax ration was 0.04. Note the congruency between the responses to the two stimuli: Smax for linear acceleration (in C) was directed along the inter-aural axis out the right ear and Smax for tilt (in D) occurred at pure roll with right ear down. Similarly, Smin for linear acceleration occurred in forward or backward movements and Smin for tilts occurred in pure nose down or up pitches.

**FIGURE 4:**
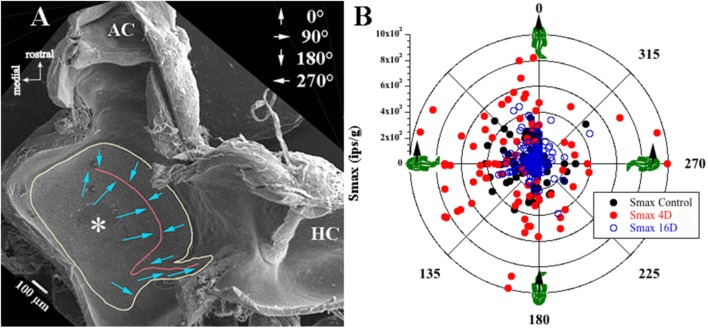
Sampling bias consideration. **(A)** Low magnitude scanning electron micrograph of the left utricular macula was flipped 180° to correspond to the right utricle for comparison with data in **B**. View from above rostral is up and medial is left (see key in upper left corner). Utricular macula is outlined in yellow, the reversal line in striola region is in red; more details of the utricle morphology can be found in Boyle et al. (2018). From left to right (medial to lateral): large medial extrastriola (asterisk), a striola zone on either side of the reversal line (red line), and a relatively narrow lateral extrastriola region approaching the ampullae of the anterior (AC) and horizontal (HC) semicircular canals. Hair bundle on each hair cell is morphological polarized and broadly depicted as blue arrows pointing from the shortest stereocilia to the kinocilium, and the polarization flips at the reversal line (red line). **(B)** Afferent responses (ips/g) are plotted in a polar graph as a function of head angle (°) at Smax for control fish and for fish exposed to 2.24 g for 4 or 16 days. Head angle at Smax of recorded responses was consistent for all fish, indicating no evidence of sampling bias from one region to another or between experimental groups. Right-hand rule was used, and 0° is + acceleration out the fish’s snout, 90° out the ipsilateral (right) ear, 180° out the fish’s tail, and 270° out the contralateral (left) ear (depicted in upper right corner in **A**). Also note that the majority of afferents recorded in these three separate conditions showed responses aligned to innervating hair cells in the medial extrastriola.

Afferent responses presented in Figure 3 are representative of 162 afferents recorded in 12 control fish, and Table 1 summarizes the principal findings (bottom row). Averaged Smax was 2103 ± 1314 ips/g (or 2.02 ± 1.39 ips/cm/s^2^) and directional selectivity was tightly tuned, having an Smin/Smax ratio of 0.085 ± 0.14 (range 0.0–0.48l, *n* = 81). Value in parentheses in Table 1 is number of afferents in which a clear Smin was also captured. One-half (*n* = 81 out of 162) of control records had a distinct Smin. To be included in the calculation of Smax at least five or more tests covering 75–90° head angles were tested and a clear maximum on a cosine function was evident. Often Smin was captured, but the recording was compromised before Smax response was reached and thus omitted from analysis. Figure 4 shows the topographical structure of the utricular macula (A) and distribution of recorded head angle at its Smax for the 162 control afferents (filled black circles) in a polar plot. Head angle at Smax occurred at virtually all angles in 2D space (±180°) in control fish, averaged 95 ± 82° (Table 1) and represents an acceleration directed out the ipsilateral (right) ear as expected for prevalence of afferents supplying the large medial extrastriola (asterisk) macula evident in Figure 4A.

**Table 1:** Physiological characteristics of toadfish utricular (Utr) afferents to sinusoidal translation acceleration as a function of duration and magnitude of exposure to HG and readaptation to 1 g.

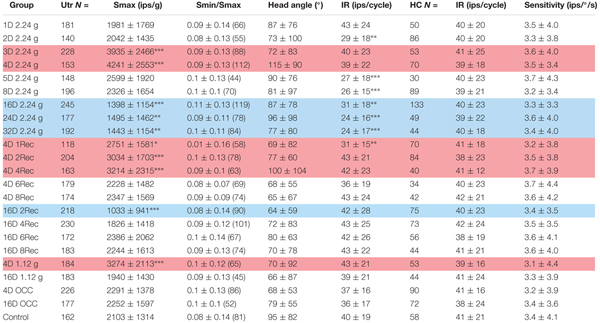

**Twenty-three (23) experimental groups (rows) were tested to controlled translational acceleration in the Earth horizon plane. Nine groups are identified by the duration of exposure, e.g., 1D indicates one (1) day (D) of exposure, to a constant resultant centripetal acceleration of 2.24 g; two groups of either 4 or 16 days of exposure to 1.12 g; five groups of 4 days of exposure to 2.24 g followed by 1, 2, 4, 6, or 8 days of recovery (Rec) in normal 1 g; four groups of 16 days of exposure to 2.24 g followed by 2, 4, 6, or 8 days of recovery in normal 1 g; two groups are identified as OCC by either 4 or 16 days of exposure to 38 revolution per minute without an appreciable centripetal acceleration; and the last group is standard laboratory control fish. Columns provide the number of utricular afferents in each group (total of 4233 afferents); response Smax of response at the afferent’s preferred direction (in impulse/s/g, or ips/g); Smin of response over the Smax as a measure of directional selectivity (number in parentheses gives the number of afferents having a verified Smin); head angle (in degree, °) at Smax; and IR at Smax (in impulse per second per cycle time). Last three columns are data obtained from number of HC afferents (*N* =), their average IR per cycle time of yaw rotation, and their averaged response sensitivity in ips/°/s of angular yaw rotation. Rows highlighted in red are early significant hypersensitivity (Smax) responses and adaption rate, and row highlighted in blue are later significant hyposensitivity (Smax) responses and adaption rate. Values are mean ± SD. Significance (ANOVA): ^∗^*p* < 0.05, ^∗∗^*p* < 0.005, or ^∗∗∗^*p* < 0.0001.**

**FIGURE 5:**
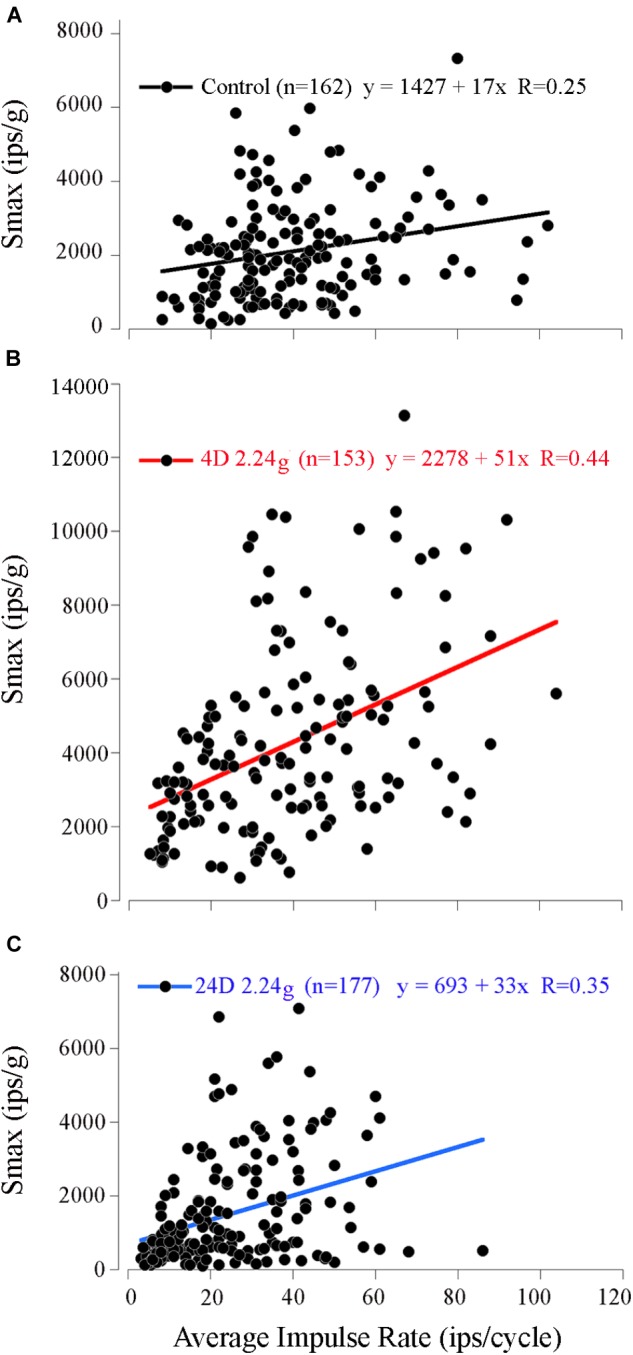
Correlation of response sensitivity and discharge properties in fish exposed to different levels of HG. A positive and significant (*p* < 0.001) correlation persisted between Smax (ips/g) and average IR at Smax (ips/cycle) for the 162 control afferents **(A)**, the 153 afferents tested after 4 days at 2.24 g **(B)**, and the 177 afferents tested after 16 days at 2.24 g **(C)**.

Measured values of Smax (range 149–7326 ips/g) and IR (range 8–102 ips/cycle; mean 40 ± 19 ips; *n* = 162) covered a broad range among control afferents. Despite the variability a weak, but significant, positive correlation was found between the Smax and IR (Figure 5A; paired correlation, *R* = 0.25; *n* = 162). Control mean IR (MIR) at rest had an even weaker, but nevertheless significant, negative correlation (paired correlation, *R* = -0.11; *n* = 107) with its degree of regularity of interspike interval as measured by coefficient of variability or CV (mean 0.53 ± 0.22; range 0.1–1.4; *n* = 107). As a result, Smax was positively correlated with CV (paired correlation, *R* = 0.28; *n* = 107), and thus broadly speaking an afferent having a high sensitivity to acceleration, a high IR during acceleration and rest, and a more irregular spacing of its interspike intervals at rest.

**FIGURE 6:**
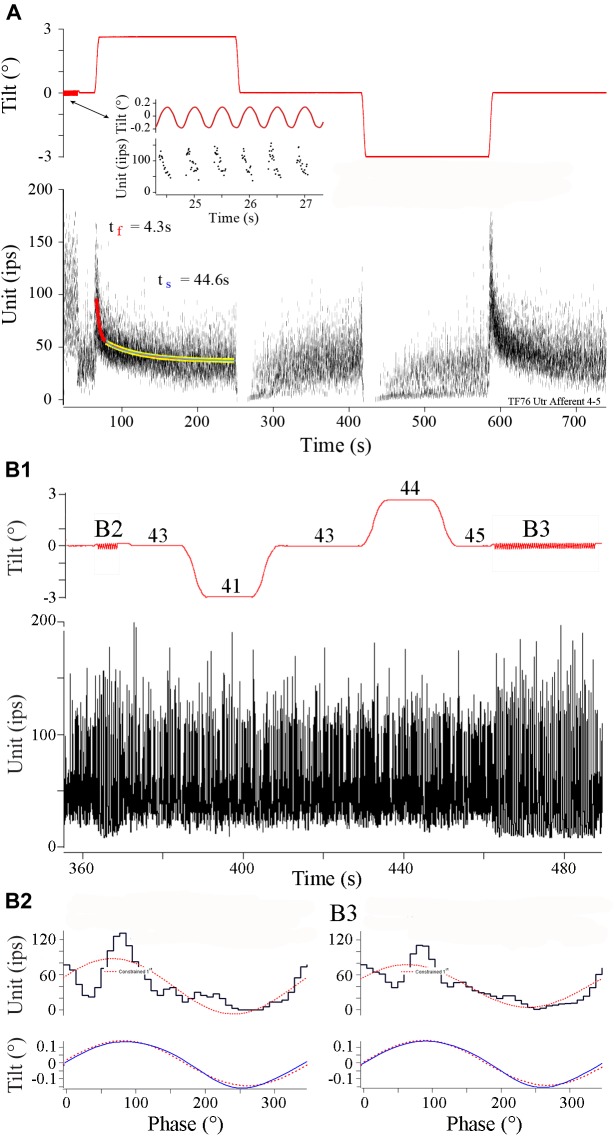
Different afferent behaviors to tilt in control fish. **(A)** Robust responses to both dynamic and static tilt displacements in control afferent. Upper trace is tilt displacement (in °) and lower trace is instantaneous IR (ips) over a continuous recording of ∼750 s. Insert shows an expanded portion early in the record during 2Hz, ±0.16°, sinusoidal tilt. Tilt stimulus was delivered at a head angle of 345°, or nearly a pure roll stimulus, with right side (positive values) down and ipsilateral to recorded afferent. Static displacements were delivered at the same head angle starting with right side down and hold, a tilt in the opposite direction to normal horizontal position and hold, a left side down and hold, and a return tilt in the opposite direction to normal horizontal position and hold. Fast (red curve) and slow (yellow curve) time constants of return to baseline IR were seen in the excitatory direction of tilt. Tilt stimuli in the opposite direction, contralateral to recording site, silenced the afferent in both instances. **(B1)** Different control afferent has a response to dynamic, but not static, tilt displacements. Same format as **A**. Sinusoidal 2 Hz tilts (±0.14°) were delivered before and after ±3° static displacements and are marked **B2** and **B3**. Phase histograms in panels **B2** and **B3** show the corresponding averaged modulation of 332 and 260 ips/°, and peak modulation lead position by 9.4 and 17.4°, respectively, respectively. During static displacements at the afferent Smax of 135°, midway between left side down roll and tail down pitch, afferent maintained a near constant IR of 41–45 ips (labeled on tilt trace).

Figure 6 shows two afferent responses to static and dynamic tilt. Afferent in panel A is a continuation of record of the same afferent illustrated in Figure 3. At the beginning of the record (see arrow pointing to insert) sinusoidal tilts at 2 Hz, ±0.16°, were delivered over seven cycles at a head angle of 345°, within 22° (and 15° from pure roll) from the calculated head angle at Smax, and the afferent showed a modulation of ±112 ips, or a unit gain of 680 ips/°, that peaked at 43°. After ∼25 s of rest the fish was tilted 3° right (ipsilateral) side down. As expected from the response to dynamic tilts, IR was elevated by ∼125 ips and showed a fast and slow time course of response decay of 4.3 and 44.6 s, respectively. Returning the fish back to initial horizontal position (near the 250 s mark) and then tilting it from the horizontal position (near the 425 s mark) to (-) 3° left (contralateral) side down suppressed the IR that recovered back near rest in a similar manner. A right-down tilt back to original horizontal position produced a comparable excitatory response.

Although responses to sinusoids and static tilts corresponded well in most tested afferents (*n* = 22), there was considerable variability in response to static tilt in nearly the same number of afferents (*n* = 18) that were responsive to translational accelerations. An extreme example is given in Figure 6B. In B1 sinusoids were presented at 2 Hz (±0.14°) both before (labeled B2) and after (labeled B3) a similar sequence of ±3° tilts in both directions spaced by returns to normal horizontal position. The fish was oriented at a head angle of 135°, and thus tilts were delivered midway between roll and pitch, corresponding to afferent Smax for dynamic tilt. Numbers associated with each step orientation ranged from 41 to 45 ips over the selected period, indicating the afferent was unresponsive to ±3° static displacements but responsive to ±0.14° displacements when presented as sinusoids. Because of this variability in tilt responses, comparisons within and across the HG experimental groups were based on afferent responses to translational acceleration.

### Control Responses to Efferent Vestibular System (EVS) Stimulation in *Normal Gravity*

Utricular afferent response to EVS activation was in general comparable to that observed in HC afferents in the same species (Boyle and Highstein, 1990b): elevation in IR and reduction in response sensitivity. As mentioned in the section “Materials and Methods,” we found that EVS activation was less prevalent in utricle than in HC recordings. To ensure efficacy of stimulation within each experiment we first recorded HC afferents to adjust placement and intensity of EVS pulses. Of the 112 HC afferents recorded and tested to EVS stimulation, only 13 (12%) were unresponsive. Table 2 gives the action of EVS stimulation on IR of control utricular afferents (bottom row). Of the 32 afferents in which EVS activation could be paired to the same afferent’s IR at rest only ∼one-third (37%) were responsive whereas 63% of the afferents were unresponsive in the same preparations as the HC recordings. The effective action of EVS stimulation on utricular afferents was a near doubling of IR from 37.1 ± 23.7 to 62.8 ± 30.5 ips, without altering CV (0.6 ± 0.32 at rest and 0.58 ± 0.31 during EVS excitation).

**Table 2:** Physiological response of utricular afferents to electrical activation of efferent vestibular system (EVS).

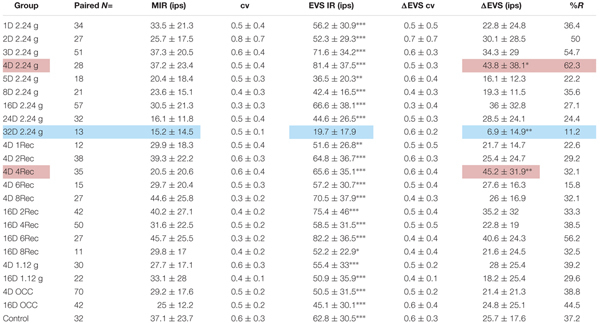

**Same groups are given in Table 1. Columns provide the number of afferents in each group with paired control and EVS responses; the MIR at rest (ips); coefficient of variability (cv) at rest as a measure of the degree of regularity of interspike interval; MIR (ips) during EVS stimulation; cv measure during EVS stimulation; absolute change in IR during EVS stimulation above IR at rest; and the percent change in IR response (%R). Red highlights the significant elevation of the IR during EVS stimulation; blue highlights the significant depression of the IR during EVS stimulation. Significance (ANOVA): ^∗^*p* < 0.05 or ^∗∗^*p* < 0.005.**

**FIGURE 7:**
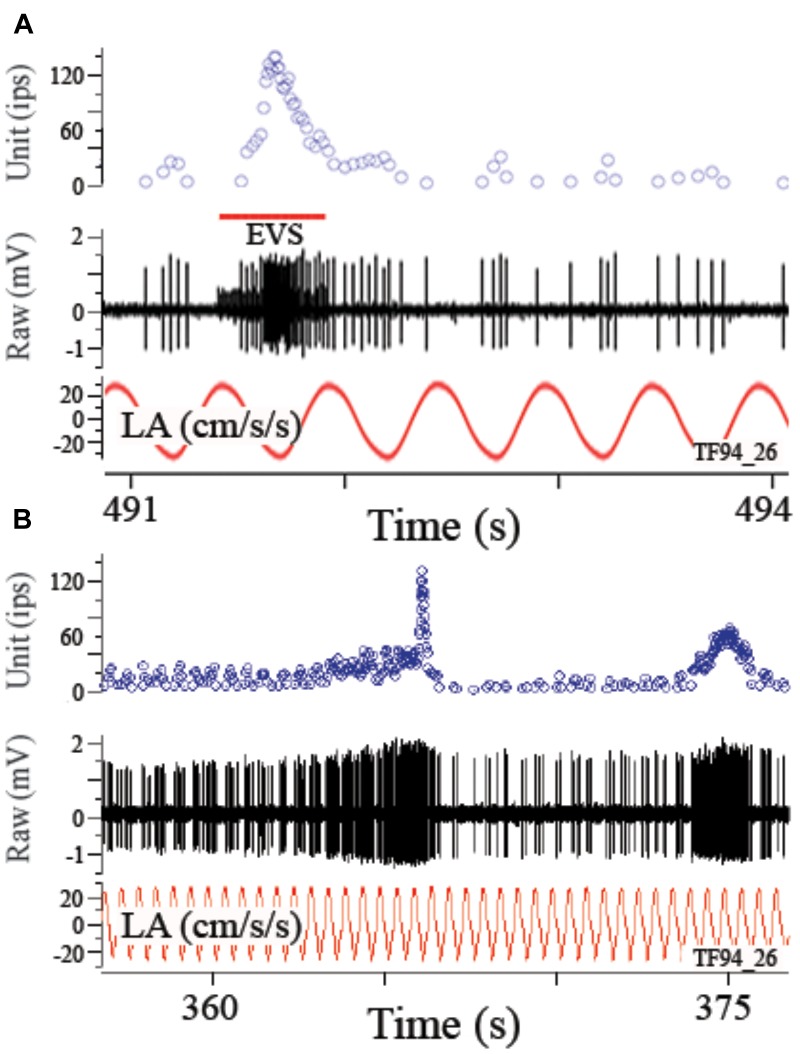
Electrically evoked **(A)** or self-generated **(B)** efferent vestibular system (EVS) activation elevates afferent rate and reduces or even blocks its response to linear acceleration. In the absence of EVS activation afferent showed an averaged response of 2 Hz linear acceleration. During a brief pulse train of EVS stimulation **(A)**, afferent IR was elevated and its response to applied acceleration disrupted. This result is also seen when the fish activated the EVS on its own as seen in the two epochs in **B**. Self-generated epochs of EVS activation excite the afferent with differing patterns and alter its response to an applied perturbation. Traces in **A** and **B** are from top to bottom instantaneous firing rate (Unit, ips), EVS pulse train, raw voltage of afferent (mV), and the applied linear acceleration (LA, in cm/s^2^).

**FIGURE 8:**
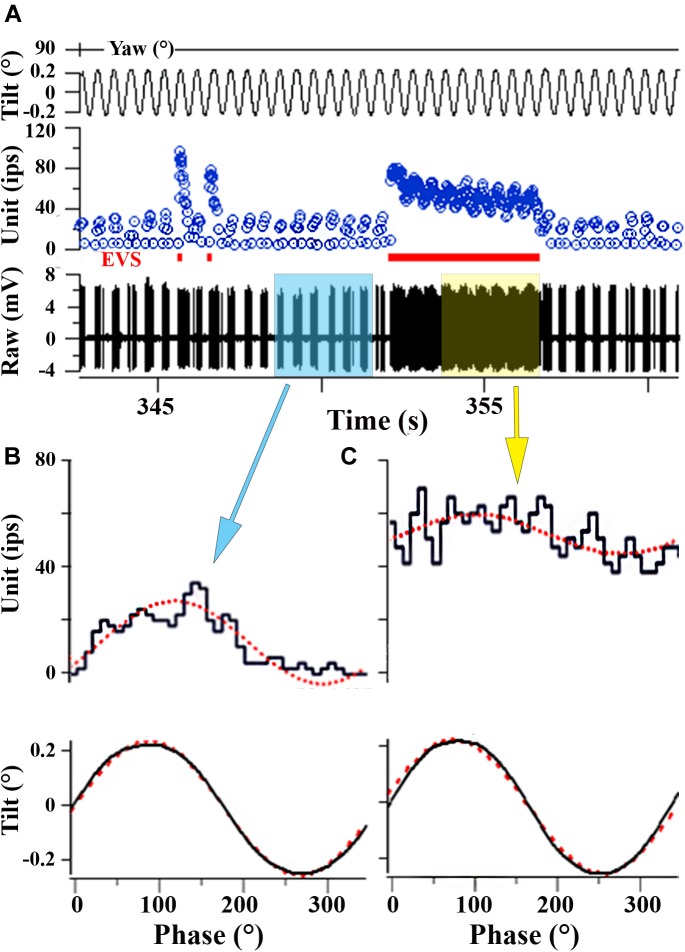
EVS activation elevates IR and partially shunts afferent response to linear acceleration. **(A)** Afferent response to 2 Hz (±0.24°) sinusoidal pure nose-down pitch in absence and presence of electrical EVS stimulation. Segment of record without EVS stimulation (time ∼350 s) is highlighted in blue box in **A** and the corresponding averaged response histogram is given in **B**; a similar portion of record in **A** is captured during EVS stimulation and highlighted in yellow box and its corresponding averaged response histogram is given in **C**. **(B)** During control cycles, afferent responded with a ±15.5 ips modulation about a discharge rate of 11.6 ips, and averaged ±63.6 ips/° and a phase lag re: position of –39°. **(C)** During cycles with combined EVS stimulation, modulation dropped by ∼50% to ±7.4 ips, now centered about an elevated discharge rate of 51.9 ips, and averaged sensitivity was reduced by about one-half to 31.4 ips/° and the phase lag (–34°) remained unaffected in the selected portion of the record. Traces in **A** from top to bottom: position of yaw axis at 90° during tilt stimulus corresponding to roll tilt, instantaneous firing rate (in ips), EVS epochs, and raw voltage of afferent (in mV). **(B, C)** Averaged responses from selected portions of the record in **A**. Ordinates in **B** and **C** are equivalent to show the elevation in discharge rate and reduction of response modulation (see text for further explanation).

Afferent modulation to a linear acceleration can be profoundly influenced by EVS activation. Figure 7 shows disruption of response to a 2 Hz sinusoidal linear acceleration of ±24.4 cm/s^2^ (or ±0.025 g) by EVS activation via a shock train applied to the brainstem (A) or self-generated by the fish itself (B). In the absence of EVS activation the afferent had an averaged response modulation of ±6.3 ips (±0.26 ips/cm/s^2^ or ±252 ips/g) centered about an IR of 7.2 ips that was temporarily altered by the brief epoch of EVS stimulation. When EVS action was less pronounced, afferent rate was elevated while at the same time its response modulation was decreased but not abolished. Record in Figure 8A shows instantaneous IR (unit, in ips) and raw voltage record (in mV) for 2 Hz ± 0.24° sinusoidal tilt of the fish rotated 90°, thereby delivering pure roll (±right down/left up) stimuli. EVS stimulation elevated IR 4.5-fold from 11.8 ips (B, control cycles) to 51.9 ips (C), while halving response modulation from 11.6 ips (or 63.6 ips/°; B, control cycles) to 7.4 ips (or 31.4 ips/°; C). Mean percent reduction in response sensitivity to linear acceleration in 27 tested utricular afferents was 56.7 ± 24.1 (range 18–88%; afferents in which the response was completely abolished were not included in this calculation). During a more steady state level of EVS activation after the initial onset of EVS pulses, the temporal relation of response modulation to stimulus (phase in ° with respect to peak °) was unaffected, similar to that seen in HC afferents (Boyle and Highstein, 1990b). However, the response to acceleration over any portion of the stimulus cycle can be dramatically altered during short bursts of EVS activation, such as the records in Figures 7A,B and the two brief epochs shown in the first portion of record in Figure 8A.

### Utricular Afferent Responses to Acceleration in On-Center-Control (OCC) Condition

**FIGURE 9:**
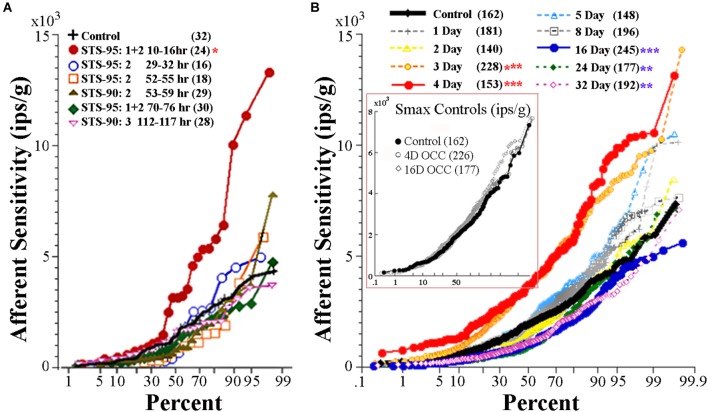
Effect of reduced and enhanced gravity on maximum response sensitivity (Smax) of utricular afferents. **(A)** Data were collected after microgram exposure on STS-90 and -95 orbital shuttle missions (modified Figure 3 of Boyle et al., 2001). Percent plot shows the afferent Smax as a function of time after landing in hours from first (10–16 h) to last (112–117 h) recording session. Within the first day after landing, Smax (red circles) to an applied linear acceleration was significantly (*p* < 0.01; ^∗^) greater than for controls (solid black line). Sensitivity returned to near normal values after ∼30 h following landing, as revealed by the data collected in the same fish at varying hours of delay after landing and indicated by separate symbols. (**B**, insert) OCCs at 4 and 16 days of exposure to 228°/s (38 rpm) constant angular velocity to mimic the rotation during 2.24 g centrifugation are plotted in four fish each together with non-rotated control afferents. Smax was indistinguishable in the three control groups and revealed no influence of rotation alone on response behavior of utricular afferents (insert). **(B)** Mean Smax of control afferents to a standard translation is 2103 ± 1314 (*SD*; *n* = 162) ips/g, and their responses lie on the solid black trace in percent plot. Afferent responses within each group for designated days of 2.24 g (1 day, 2 days, …) exposures are given with separate symbols and traces as indicated in the key. A significant elevation of Smax at 3-day (*n* = 228 afferents) and 4-day (*n* = 153 afferents) was observed (*p* < 0.0001; ^∗∗∗^); 90–100% of the afferents in these groups had a markedly greater Smax than the 162 control afferents. The elevation was followed by several days (5 to ≥8 days) of normal afferent sensitivity and then by a significant decrease at 16-day (*n* = 245 afferents; *p* < 0.0001; ^∗∗∗^), 24-day (*n* = 177 afferents; *p* < 0.005; ^∗∗^), and 32-day (*n* = 192 afferents; *p* < 0.005; ^∗∗^). Number of afferents recorded in each group is given in parentheses.

During centrifugation, the fish was exposed to both a centripetal force dependent on its eccentricity and constant rotations per minute (rpm) and the constant force of gravity. A separate device was used to place the fish precisely at the center of rotation thereby matching as well as possible all parameters except now in the absence of centripetal force. Fish were exposed to either 4 or 16 days of rotation at 38 rpm. As listed in Table 1, the OCC utricular afferents behaved statistically identical in all measured parameters after both 4-day (*N* = 226 afferents) and 16-day (*N* = 177 afferents) to the control fish not exposed to any experimental condition prior to recordings. In the insert in Figure 9B Smax (in ips/g) of individual responses are plotted as a function of percent within each control group, and shows the complete overlap of OCC afferents with their control counterparts.

### Horizontal Canal Afferent Behavior in *Normal and Altered Gravity*: Internal Control

Horizontal canal afferents recorded in control fish in normal gravity (*n* = 58) behaved in an identical manner to that previous reported in toadfish (Boyle and Highstein, 1990a). Table 1 (right columns) gives the consistent averaged IR during sinusoidal yaw rotation (in ips/cycle) for 58 control HC afferents, 162 HC afferents in On-Center-Control (OCC) afferents, 604 HC afferents recorded after 1–32 days of 2.24 g centrifugation, 518 HC afferents recorded after 4 or 16 days of centrifugation followed by a recovery period, and 97 HC afferents recorded after 4 or 16 days of 1.12 g centrifugation. Despite the wide range of duration and intensity of the experimental conditions, HC afferent IR was unaffected.

Horizontal canal afferent responses to yaw rotation were also unaffected by the experimental conditions (*q.v.* Supplementary Figure 1). HC afferents exhibit a wide range of response dynamics and can be broadly termed as low or high gain velocity encoding and acceleration encoding afferents (Boyle and Highstein, 1990a), and the prevalence of each afferent population and its response characteristics were equivalent under all experimental conditions. Table 1 (right columns) gives the averaged response sensitivity (in ips/°/s) of HC afferents to a 0.5–1 Hz (±10–15°/s) sinusoidal angular yaw rotation for each experimental group. No significant difference was observed in response and IR to rotation among the recorded afferents, suggesting there was no broad, non-descript action on the periphery during the experimental tests. In conclusion, behavior of HC afferents serves as an internal control of variables associated with centrifugation, such as the duration (1–32 days) of 1.12 or 2.24 g centrifugation, length of recovery to normal gravity (1–8 days), and constant angular rotation without centripetal force (OCC) used in the present study.

### Utricular Afferent Responses to Acceleration and Tilt Following 2.24 g Exposure

Principal reaction to HG was an adjustment of *response sensitivity* as a function of the magnitude and duration of exposure (*q.v.* Supplementary Figure 2). Reaction was bi-phasic starting with a significant twofold *increase* in Smax after 3 (*N* = 228 afferents) and peaked at 4 (*N* = 153 afferents) days of exposure (highlighted in red in Table 1), with a return to normal values after 5 (*N* = 148 afferents) and 8 (*N* = 196 afferents) days of exposure, and then followed by a significant *reduction* by ∼one-third after 16 (*N* = 245 afferents), 24 (*N* = 177 afferents), and 32 (*N* = 192 afferents) days of exposure to 2.24 g (highlighted in blue in Table 1). The Smin/Smax ratio remained the same on average for all populations within and across the wide range in experimental conditions. Interestingly, significant changes were observed in average IR at Smax (in ips/cycle; Table 1): average IR was reduced at 2D prior to the sensitivity increase at 3D, and stayed at a 20–40% lower level from 5D to 32D despite the significant changes in Smax over these periods of 2.24 g exposures. Records from the 4- and 16-day fish confirmed that the Smax of the majority of afferents, like their control counterparts, occurred at head angle associated with a positive acceleration directed out the ipsilateral labyrinth (Figure 4B, Table 1). In addition, the positive correlation of Smax and average IR (Figures 5B,C) was maintained after exposures to HG, even with a significant reduction in both Smax and average IR recorded in the 16-day fish (Table 1). Correlations within each group between MIR (ips) and CV and, in turn, Smax were also maintained at the same level of confidence. Data obtained in recovery periods to 1 g from 1 to 8 days after HG indicated that average IR set point was restored after 2 days in normal gravity. It is important to note that HC afferents recorded in each experimental group not only were unaffected in terms of response modulation to yaw rotation as mentioned above, but also had an average IR (41 ± 21 ips/cycle) equal to that of utricular control afferents (40 ± 19 ips/cycle) that remained consistent under all experimental conditions (right two columns of Table 1).

### Utricular Afferent Responses to Acceleration Following 1.12 g Exposures

In a separate, and smaller, sample afferents were studied after 4 or 16 days of HG at one-half the resultant force as the larger sample. A significant elevation in Smax (3274 ± 2113; *N* = 184 afferents; *p* < 0.0001, ANOVA) was observed after 4-day exposures to 1.12 g (highlighted in red in Table 1), about half way between control Smax and 4D 2.24 g Smax. Although a reduction in Smax was seen on average in 183 afferents studied after 16-day exposures, it was not statistically significant. All other parameters of response to acceleration and IR properties were unchanged.

### Comparison of Utricular Afferent Responses to Acceleration Following Micrograms and 2.24 g Exposures

Utricular afferents exhibited a profound hypersensitivity when tested to translation upon return to Earth after 9–16 days of microgram exposure on two shuttle orbiter missions and a recovery back to normal after several days (Boyle et al., 2001). These data are given in the percent plot in Figure 9A. Nearly 60% of the afferents tested within 10–16 h after landing (solid red circles) were significantly (*p* < 0.005) more sensitive than control afferents (solid black line). To put this in perspective an afferent for example had a near saturating IR modulation of ±30 ips for a 1.6 Hz stimulus at an amplitude of ±0.0026 g, in essence a minute vibration that otherwise would be mostly ineffective in eliciting a modulation.

**FIGURE 10:**
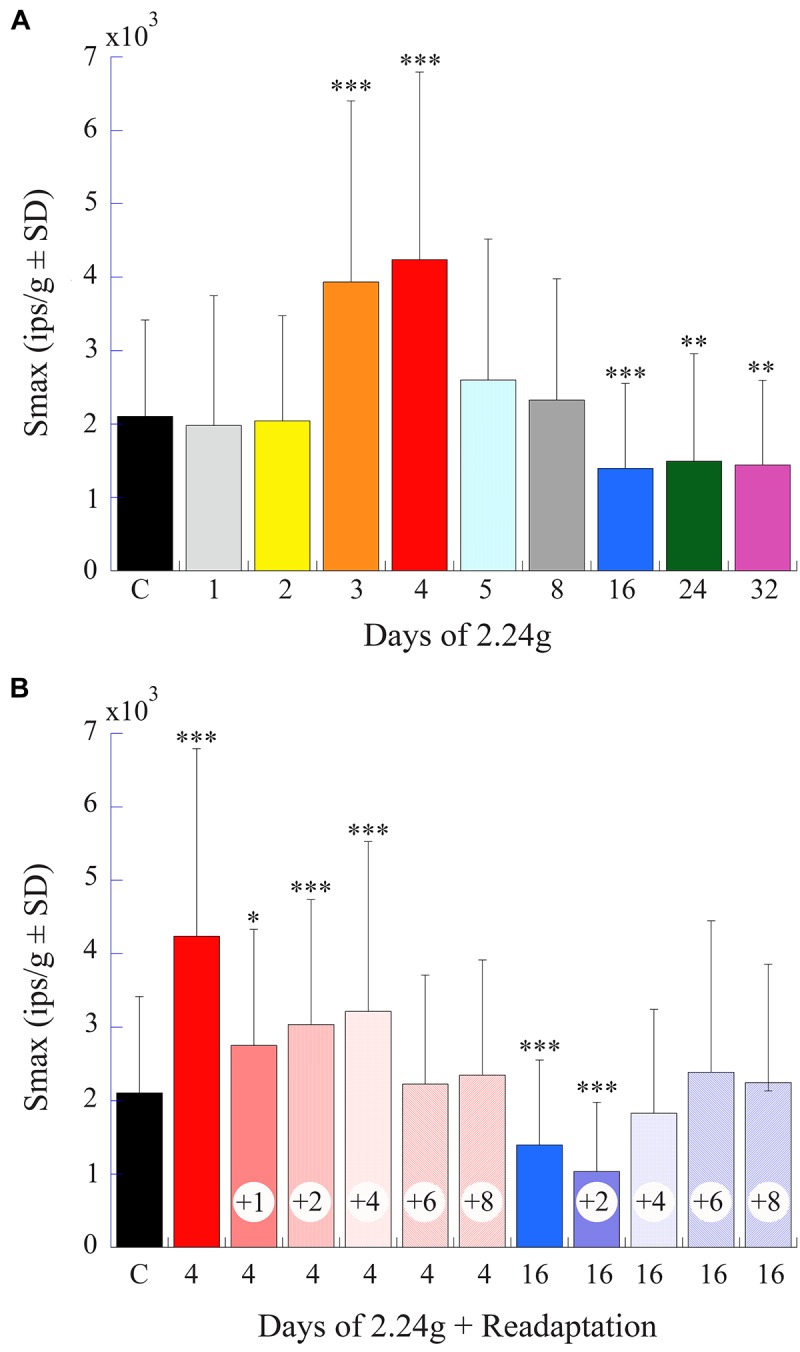
Afferent Smax as a function of days of exposure to 2.24 g and readaptation after return to normal 1 g environment. **(A)** Histogram plots the mean afferent Smax (ips/g ± SD) (same data as shown in Figure 9B) against time of exposure to centrifugation (in days). **(B)** Histogram plots afferent Smax as a function of number of days (indicated by number inside of each column) in normal 1 g after 4- and 16-day exposures to centrifugation to the return to baseline levels. Initial hypersensitivity recorded immediately after 4-day exposures (solid red unlabeled column) required >4 days to recover to control levels. The later hyposensitivity observed after 16-day exposures (solid blue unlabeled column) required at least 2 days to recover. In each panel error bars are ±*SD*, the left column labeled **C** is the control response values, and asterisks designate the level of significance of *p* < 0.05 (^∗^), *p* < 0.005 (^∗∗^), and *p* < 0.0001 (^∗∗∗^) to control measures.

Figure 9B gives a percent plot of afferent Smax to translation for control fish (solid black line and insert) and for those after exposure to varying duration of 2.24 g from 1 to 32 days in a comparable manner to Figure 9A for comparison. Initial hypersensitivity was most evident in afferents after 3 (solid orange circles) or 4 (solid red circles) days of 2.24 g exposures, and the separation of Smax from control population was nearly 100%. Responses in these exposures unmistakably resembled those seen initially after space travel and were unexpected. Smax recovered within normal levels at the 5- and 8-day tests before a significant Smax reduction was observed after 16–32 days of 2.24 g exposures (Table 1). Mean Smax (ips/g ± SD) of the experimental data presented in Figure 9B are plotted against days of exposure to 2.24 g in Figure 10A. Interestingly, afferent characteristics across the three (16-, 24-, and 32-day) groups displaying a Smax hyposensitivity was statistically similar (ANOVA), suggesting a plateau had been reached at this stage of the adaptation process.

### Re-adaptation of Afferent Response After Return to Normal Gravity

Figure 10B provides a summary histogram (same format as Figure 10A) of Smax in afferent populations initially exposed to 4 or 16 days of 2.24 g and returned to 1 g until a restoration of normal activity was established. Early hypersensitivity observed after 4 days at 2.24 g took >4 days to recover to control levels, and the later hyposensitivity observed after 16 days at 2.24 g recovered more quickly and required >2 days in 1 g to recover to control levels. Thus, the bi-phasic hyper- to hypo-sensitivity to translations after centrifugation reflected an adaptive process that was restorable with a delay to its original state.

### Possible Mechanism(s) of Afferent Adaptive Processes to Altered Gravity: Efferent Action

By inference afferent sensitivity is expected to lessen or IR to rise or both when EVS is activated episodically or in a steady state; conversely, the removal of a steady state EVS action could lead to an afferent sensitivity increase and IR could remain the same or even decrease. Comparing the results in Table 1 it could be inferred that the early increase and later decrease in Smax was due to an OFF and On EVS action on the hair cell alone, respectively, without a direct action on the afferent. EVS action on afferent rate was almost exclusively excitatory (Figures 7, 8) from the axo-dendritic synapse on the afferent and not through the axo-somatic synapse on the hair cell (Boyle et al., 2009). No consistent evidence emerges from pairing Smax and IR to support a direct systematic EVS role on afferent processing after centrifugation.

Table 2 tabulates the magnitude and prevalence of response of EVS activation for paired data within each test group. With only one exception, EVS pulses significantly elevated as expected the IR (EVS IR) above the MIR of background IR in all groups. The sole exception was the afferent group subjected to 32 days at 2.24 g (32D 2.24 g). Here, afferent response to EVS activation was noticeably infrequent with only 11.2% responding (against 37.2% in control afferents), and noticeably weaker with a difference of EVS to non-EVS epochs or IR (ΔEVS) significantly less (6.9 ± 14.9 ips) than that for control afferents (25.7 ± 17.6 ips). These findings at the 32-day mark are not consistent with the significant Smax reduction (Table 1). In the same vein, the afferent response to EVS pulses after 4 days at 2.24 g (4D 2.24 g) was more prevalent with 62.3% responding with significantly greater ΔEVS (43.8 ± 38.1 ips) than that for control afferents. These findings suggest that EVS action was greatly enhanced at 4-day mark, but again the action is not consistent with the significant Smax increase (Table 1). At the present stage it is not possible to establish direct causality of afferent IR and response behavior after HG exposures and EVS action.

### Possible Mechanism(s) of Afferent Adaptive Processes to Altered Gravity: Synaptic Remodeling

**FIGURE 11:**
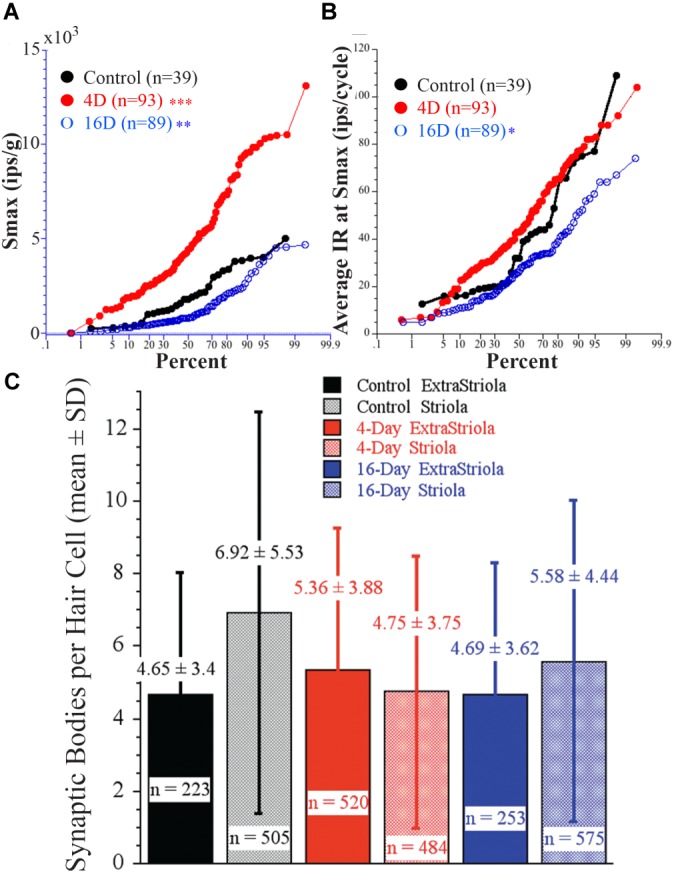
Direct comparison of *physiology* of afferents as a function of duration of exposure to 2.24 g to *synaptic organization* of hair cells in the *same* fish. **(A)** Percent plot of Smax shows significantly higher Smax in 4-day (4945 ± 2823 SD imp/s/g; *n* = 93) and significantly lower Smax (*p* < 0.002) in 16-day (1245 ± 1097 SD imp/s/g; *n* = 89) fish from controls (2045 ± 1314 SD imp/s/g; n = 39). **(B)** The average IR (in ips/cycle) during acceleration at Smax also differed between afferents in these groups: rate was significantly lower in the 16-day (28 ± 15 SD ips/cycle), and higher, but not significantly so, in 4-day (46 ± 21 SD ips/cycle) fish from the control afferents (39 ± 24 SD ips/cycle). Level of significance (ANOVA): *p* < 0.0001 (^∗∗∗^), *p* < 0.002 (^∗∗^), and *p* > 0.01 (^∗^). **(C)** Number of synaptic bodies per hair cell was determined using 3D computerized serial reconstruction techniques at transmission electron microscopy level from 150 sections of 180 nm thickness from two macular regions, one straddling the reversal line (shaded columns) and the other 150 μm away in the medial extrastriola (solid columns), in control fish (black) and fish exposed to 2.24 g for 4 (red) and 16 (blue) days. Average (±*SD*) number of synaptic bodies is given above each column. Number (*n* =) of completely reconstructed hair cells used in this analysis was large and ranged from 223 to 575 hair cells as indicated. Variability within each group was very large and thus, despite the high number of reconstructed hair cells, SB densities in the two macular regions were NOT significantly different between control and centrifuged fish despite the highly significant differences in their responses to acceleration.

Afferent response sensitivity and hair cell synaptic organization were examined in the *same* animal to determine the possible causality between response magnitude and number of synaptic bodies. Figure 11 shows percent plots of Smax (A, in ips/g) and average IR (B, in ips/cycle) in three groups: controls (black solid circles and line; *N* = 2), 4 days at 2.24 g (red solid circles and line; *N* = 2), and 16 days at 2.24 g (blue open circles and line; *N* = 2). Importantly, highly significant differences in response sensitivity were found in the same fish from which SB counts were taken. In Figure 11A, Smax was significantly higher (*p* < 0.0001, ANOVA) in 4-day (4945 ± 2823 SD imp/s/g; two fish; *n* = 93) and significantly lower (*p* < 0.002, ANOVA) in 16-day (1245 ± 1097 SD imp/s/g; two fish; *n* = 89) fish from controls (2045 ± 1314 SD imp/s/g; two fish; *n* = 39). In Figure 11B, the average IR at Smax (in ips/cycle) also differed: rate was significantly lower (*p* < 0.01, ANOVA) in 16-day (28 ± 15 SD ips/cycle), and higher, but not significantly so, in 4-day (46 ± 21 SD ips/cycle) fish from control afferents (39 ± 24 SD ips/cycle). Figure 11C gives the results of hair cell SB counts in the *same* fish from which afferent responses were obtained (Figures 11A,B). Number of SB per 100 μm^2^ was determined for two macular regions, one straddling the reversal line in the striola (shaded columns) and the other 150 μm away in the medial extrastriola (solid columns), in controls (black) and fish exposed to 2.24 g for 4 (red) or 16 (blue) days. On average SB count per hair cell was 4–7 in the two macular regions in each experimental group, and ranged in number from 1 to 13 (*n* = 223) in control medial extrastriola, 1 to 25 (*n* = 505) in control striola, 1 to 20 (*n* = 520) in 4-day medial extrastriola, 1 to 22 (*n* = 484) in 4-day striola, 1 to 16 (*n* = 253) in 16-day medial extrastriola, and 1 to 20 (*n* = 575) in 16-day striola regions. Variability within each group was large and thus, despite the high number of hair cells completely reconstructed in three dimensions, single and multiple SB densities in the two macular regions were not significantly different between control and HG fish. This is in stark contrast to the clear separation in afferent physiology.

## Discussion

As a necessary first step in determining the role of the utricle in neural adaptation to novel gravity loads, otolith afferents were studied to establish benchmarks for comparison within and across the experimental groups. Vestibular afferents, both semicircular canal (frog: Honrubia et al., 1989; toadfish: Boyle and Highstein, 1990a; Boyle et al., 1991; bird: Landolt and Correia, 1980; chinchilla: Baird et al., 1988; cat: Anderson et al., 1978; squirrel monkey: Fernández and Goldberg, 1971; rhesus monkey: Schneider et al., 2015) and otolith fibers (cat: Loe et al., 1973; pigeon: Si et al., 1997; primates: Fernández and Goldberg, 1976a,b,c; Angelaki and Dickman, 2000; Jamali et al., 2009; Yu et al., 2012) differ from one another in their discharge patterns and response properties to adequate stimuli. As seen in other species toadfish utricular afferents showed a wide range of discharge rates, regularity of discharge, and response sensitivity to sinusoidal linear acceleration with a typically tight degree of directional selectivity.

The physical basis for differences in response dynamics among otolith afferents is not fully understood, and determining the precise signals originating from different regions of the utricular macula and how these signals enter central otolith reflex pathways have been a challenge. Afferent dendritic organization to the type of hair cell (type I and/or type II) it supplies varies in complexity (see review by Goldberg et al., 2012; Boyle et al., 2018), and it is reasonable to assume that hair cell excitation is not uniform (Spoon et al., 2011; Curthoys et al., 2017). In chinchilla utricle about three-fourths of recorded afferents displayed a more regular discharge of their interspike intervals (Goldberg et al., 1990a,b), and corresponds to about three-fourths the total area of macula designated as the medial extrastriola. Toadfish utricle is structurally similar in the broad sense to other species, including amniotes and other anamniotes, and its hair cell polarization is highly organized across the more 2D macula surface (Boyle et al., 2018). Like the chinchilla, toadfish medial extrastriola occupies about three-fourths the total macula area (Figure 4A) and reflects the responses of the majority of afferents recorded in this study (Figure 4B). Unlike the chinchilla, regularity of discharge of toadfish utricular afferents span a continuum but three-fourths of them are more irregularly discharging. An attractive hypothesis is that vestibular afferent inputs are matched to the dynamic requirements of the various reflexes they target (Highstein et al., 1987; Boyle et al., 1992; McArthur and Dickman, 2008), and evolutionary pressures drive the particular behaviors (Nilsson, 2013; Straka and Baker, 2013; Straka et al., 2014). In this scheme, the prevalent irregular utricular afferents are best matched to the control requirements of the rapid attack and escape avoidance of toadfish.

### Utricular Afferent Responses After Microgravity (μg) Exposure

From the earliest manned orbital missions we know that changes occur in processing of gravitational information during adaptation to microgram and re-adaptation to 1 g upon return to Earth. The adaptive mechanisms to altered gravity might be confined to the peripheral sensory structures or involve central processing centers or likely require the participation of both the peripheral and central nervous systems. Within the first day after STS-90 and -95 shuttle landings, a response hypersensitivity to acceleration stimuli was observed in utricular afferents (Figure 9A; Boyle et al., 2001). Reduced gravity in orbit during these relatively short microgram exposures apparently resulted in an upregulation of afferent sensitivity. Time course of return to normal sensitivity in toadfish afferents paralleled the reported decrease in vestibular disorientation in astronauts after return from shuttle missions (Reschke et al., 1994). Although belonging to a distinct group of animals, recordings taken directly from gravitoreceptors of the land snail (phylum Mollusca), and thus no intervening synapse as in afferent recordings, after exposures of 12–16 (Balaban et al., 2011) and 30 (Aseyev et al., 2017) days in space also showed a pronounced response hypersensitivity to tilt. In a complimentary study in tilapia the gain of an otolith-related vestibuloocular reflex was significantly increased within the first postflight week, and returned to control levels in the second postflight week, of space missions (Sebastian et al., 2001).

Orbiting space stations provide a platform to extend human presence in space and capture the physiological response(s) to longer duration of exposures. Hallgren et al. (2016) measured in 1 g the ocular counterrolling (OCR), an otolith-mediated reflex, during centrifugation before and after a 6-month stay in space in 25 astronauts and found a significant reduction in OCR magnitude after landing and its restoration to normal levels in 9 days. Their results are largely in agreement with changes in gaze control reported by Kornilova et al. (2012) in cosmonauts to 129–215 days in space. Adaptation to microgram is likely to change over time from an initial over compensation to restore normalcy to a hyposensitivity more in line with the prevailing gravity. Do the processes of adaptation to microgram in orbit or weightlessness in deep space missions reach a plateau or continue to evolve in time are questions yet to be answered.

### Utricular Afferent Responses After Hypergravity Exposure

Based on the robustness of postflight hypersensitivity of utricular afferents, we expected to find a compensatory *hyposensitivity* in response to HG. The initial hypersensitivity was unexpected and developed at Day 3 and peaked at Day 4. It was followed by a period of normalcy and then the development of the anticipated hyposensitivity after 16–32 days of HG. Initial hypersensitivity was robust and recovered to normal levels after ∼4–6 days at 1 g. Utricular afferent response to HG did not reflect a systemic action on the labyrinth as a whole, since HC afferents were consistently normal in response and discharge properties and remained independent of level and duration of HG in the *same* fish. OCCs performed at 4- or 16-day exposure to 228°/s (38 rpm as in 2.24 g runs) constant angular velocity were identical in all measures to their utricular and canal afferent counterparts in non-rotated controls. Video taken during the light cycle did not reveal any aberrant behavior during the different exposures of 2.24 g, perhaps suggesting a central compensatory mechanism in operation. Of prime importance is the fact that in the same species we have the neural response to microgram and a more comprehensive evaluation of the neural response to HG. These results demonstrate that the utricle undergoes continuous plasticity based on magnitude of the prevailing gravity and suggest the possibility that the process of re-adaptation from HG exposure might mimic the process of adaptation to microgram.

The organism’s response to transitions in gravity is not simply a step increase or decrease, but varies with time of exposure to the new level of force. Majority of astronauts experience mild-to-severe disorientation for 3–4 days upon initial exposure to microgram, a condition akin to terrestrial motion sickness (Reason and Brandt, 1975). Even though most humans eventually adapt after days to microgram, return to 1 g causes vestibular dysfunction (Reschke et al., 1994; Oman, 1998; Black et al., 1999). The behavior (Anken and Rahmann, 1998) and otolith afferent response sensitivity (Boyle et al., 2001) of fish follow a comparable course of readaptation after landing and therefore restoration to normalcy is likely a general reaction of the organism to short duration (weeks) exposure to microgram. Our data (Figure 10) confirm that readaptation also occurs in afferent response to HG.

The organism’s early response to an abrupt change in the force of gravity is remarkable. In the Russian Cosmos/Bion missions many of the effects observed early in flight on vestibular and oculomotor function in nonhuman primates are consistent with response hypersensitivity. Badakva and Kozlovskaya summarized their results in Cohen et al. (2005). Within the first days in orbit gaze shifts to lateral visual targets were hypermetric, despite a reduction in head displacement. This hypermetria caused positional errors and was compensated by an increase in gain of the angular vestibulo-ocular reflex (aVOR) to 1.5 on Day 2 and remained high (2.0) up to Day 8. Associated with gaze hypermetria, response of vestibular nuclei neurons during gaze fixation task gradually increased to a maximum on Day 4, like our present HG data, and returned back to normal for the rest of the 1–2 week flight. Gualierotti and colleagues (Bracchi et al., 1975) directly addressed otolith afferent discharge in μG in technically challenging experiments in the bullfrog. Although the interpretation of their data is difficult, otolith afferent discharge appeared to increase within the first 3 days of the orbital mission and returned to normal at Day 5. Interestingly, this closes matches our HG data after exposure to 2.24 g. The initial reaction in astronauts and animals points to a possible common mechanism. Although it is clearly a speculation, the evidence suggests an exaggerated otolith reaction is evoked to a novel gravity challenge. This challenge occurs in either direction from a loss of gravity in spaceflight to an applied force by centrifugation on the ground. To what extent is this simply a transitory maladaptation or a behaviorally relevant response remains to be determined. Unfortunately, critical data in real-time targeting this initial response to microgram and HG remain lacking.

### Mechanisms of Utricular Adaption to Gravity

We sought to elucidate potential mechanisms underlying utricular adaptation to transitions in gravity levels, particularly hair cell synaptic remodeling and EVS action. Observed changes are thought to reflect the following possibilities: (a) change in transducer sensitivity, (b) temporary structural alteration affecting mechanoreception, (c) pre- or post-synaptic alteration in the strength of synaptic transmission, or (d) neural feedback circuit or efferent copy switched on or off. We examined the latter two possibilities in detail and both attempts brought us no closer to identifying a clear mechanism that initiate and regulate adaptation.

(a) Transducer changes. Prolonged deviation of the sensory hair bundle leads to an adaptation of otolith hair cell receptor potentials (Eatock et al., 1987; Goldberg et al., 2012). Manifestation of this adaptation can be seen in responses of some otolith afferents to prolonged steps of linear acceleration (Fernández and Goldberg, 1976a,b). Microgravity might be thought of as a step of hair bundle motion in the “off” direction, and adaptation in transducer mechanism(s) might negate any aberrant afferent response in microgram. The converse might occur in HG. The onset and time course of transducer adaptation might be both rapid and long-lasting, and involve a reconfiguration of the transmembrane channel (TMC)-like proteins of the transducer pore (Pan et al., 2018). Our data do not offer a clear insight.

(b) Structural changes of otolith mass. Otolith weight by gravity intensities might regulate the otoconia mass and alter its coupling to stereocilia in a manner that affects mechanotransduction, for example a change of otolith–stereociliary coupling that adjusts bundle deflection for a given movement (Fredrickson-Hemsing et al., 2012). Wiederhold et al. (1997, 2000) showed that the otolith mass in growing snails was increased by microgram exposure. A slowing in otolith growth during development was reported to occur under HG conditions in *neonate* fish (Anken et al., 2000a), and also after vestibular nerve transection again in neonates (Anken et al., 2000b). However, Lim et al. (1974) and Sondag et al. (1995) in well-controlled studies could not demonstrate any change in the otoconia Ca^+2^ content, shape, size, or distribution in adult hamsters subjected to HG. More recently, Aceto et al. (2015) found a decrease in otolith calcification after prolonged HG exposure in zebrafish, presumably the result of a regulation of carbonic anhydrase and other matrix protein productions (Anken et al., 2004; Anken, 2006). As bone is adversely remodeled during space missions (Demontis et al., 2017), calcium carbonate on the other hand might be deposited on existing otoconia to provide greater “weight” in an effort to restore the “gain” of gravity sensation; conversely, HG would lead to an ablation of otoconia mass to “rebalance” function. Our afferent recordings did not reveal any aberration(s) in discharge properties or spurious excitations except a change in response sensitivity, implying that if such a mechanism was in operation, the remodeling of the otolith mass was uniformly distributed. Although this mechanism is attractive, particularly for long-term habituation in a reduced gravity, we have no data collected in this study that directly addresses this mechanism.

(c) Synaptic plasticity. A widely considered candidate responsible in the adaptive response to microgram is plasticity of hair cell ribbon synapses (Moser et al., 2006). The findings of Ross (2000) promoted this hypothesis: number of ribbon synapses in certain type II hair cells in rat was labile, increasing by approximately 55% following exposure to microgram; type I hair cells were less affected. Toadfish vestibular epithelia possess only type II hair cells and lack the specialized nerve calyx seen in reptiles, birds, and mammals. Also in toadfish the number of *en passant* and terminal boutons of a given semicircular canal afferent, indicating a potential locus for synaptic coupling to a hair cell, are positively correlated with its response sensitivity (Boyle et al., 1991). Thus, an increase in ribbon synapses in rat utricular hair cells by microgram exposure aligns with our observed post-flight hypersensitivity in toadfish utricular afferents. However, more recent evidence by Sultemeier et al. (2017) in mouse exposed to 15 days of microgram paints a different picture. They found synapse densities *decreased*, not increased as seen by Ross, in medial extrastriola hair cells and remained unchanged elsewhere in other macula regions and HC cristae. Although one species is likely more susceptible to gravity influences than another based on body mass, this discrepancy between rat and mouse is intriguing but not readily apparent, and must be resolved in future studies. As we discuss below, the picture is even more blurred when we make a direct comparison between recorded response sensitivity and 3D serial reconstruction of synaptic bodies in individual hair cells in the *same* animal.

An important piece of relevant evidence is the time of synapse formation and reformation in inner ear structures. Turnover rate of ribbon synapses in vestibular hair cells is unknown, but some estimates derived from other systems are useful. Ribbon synapses in the visual system of albino rat are said to have a life span of ∼8 h (Spadero et al., 1978) and are structurally plastic particularly in assembly and disassembly processes (Schmidt and Drenckhahn, 1993) during development (Regus-Leidig et al., 2009). In the hippocampus, asymmetric synapses form and disappear within a few minutes (Desmond and Levy, 1986). Although related to different systems, these data indicate that specific times of synapse formation are from minutes to hours (Cragg, 1969; Cotman and Nieto-Sampedro, 1982). Further, Puro et al. (1977) showed that synapses not only turn over but that the rate of turnover increases during development, suggesting that both synapse formation and termination rates are regulated and that the specificity of synaptic connections can be increased by selective termination of synapses. Thus, as sensitivity is decreased to compensate for hyperstimulation, excess synapses will be deactivated by receptor downregulation and “anchors” inside the cell mark the former receptor sites (Ziff and Barry, 2002). Since the sudden change of gravity is an extreme condition, the specific time of synapse rearrangement might be expected to be longer: from hours to several days.

Despite the attractiveness of synaptic bodies being added or subtracted as needed to regulate the system response to gravity, a key finding here is in direct opposition to this hypothesis. In the *same* fish we recorded a substantial afferent population to acceleration in control fish and in fish after 4- or 16-day of 2.24 g exposures, and conducted complete 3D ultrastructural reconstructions of hair cells in the reversal zone of striola *and* in the predominant medial extrastriola region. Afferents had highly significant differences in Smax (Figure 11A) and average IR per cycle (Figure 11B) with virtually little to no overlap. Even with this clear distinction in utricular physiology, SB counts per hair cell were statistically equivalent in the selected three afferent populations in both macular regions *measured in the same fish*: hair cells contained on average five to six synaptic bodies, but the variation in number was large (Figure 11C). Afferent Smax also showed a wide variation from less sensitive to highly sensitive within an individual animal, but were distinctly separated as a group from the other populations. The importance of this finding is that the physiological and anatomical results were obtained in the *same* animal, and no direct correlation was found. It might be argued that the utricular synaptic organization had adapted over the course of afferent recordings. We saw no indication of this: afferents after 4 and 16 days of HG were hyper- and hypo-sensitivity, respectively, from the start to the finish of the recordings and require days to readapt to 1 g. Clearly other factors might be in play. For example, adjustment of synaptic efficacy or strength might involve many processes other than the SB itself, such as Hebbian plasticity based on the correlation of pre- and post-synaptic cells (Matsubara and Uehara, 2016). Suli et al. (2016) have provided some key insights into synaptic plasticity in mechanosensory hair cells. Using transgenic and mutant zebrafish lines they found that innervation was crucial for regulating the number, size, and localization of ribbons in maturing lateral line hair cells, and for their maintenance at the mature synapse. How synaptic weights and innervation patterns might scale up or down or fluctuate in distribution within the hair cell–afferent complexes need to be examined in our model.

(d) A neural feedback circuit. It is remarkable that vertebrates exert an efferent (EVS) neural control over the sensitivity, bandwidth, and linearity of sound and motion sensation. In general, the EVS provides hair cell organs not only with the means to control passive filtering of sound and motion signals, but also to regulate the mechanical gain of the living amplifiers in the ear (Hudspeth, 2008; Rabbitt and Brownell, 2011). Efferent cell bodies are located in the caudal brainstem, a phylogenetically ancient structure that monitors and controls vital functions including alertness and arousal. A typical efferent neuron in toadfish has extensive dendritic arbors spanning the brainstem, thereby providing the neuron the capability to receive a wealth of descending command and ascending sensory information (Highstein and Baker, 1986; confirmed in the present study, *q.v.* Supplementary Figure 3). Spatial coding is likely not selective, since efferent fibers can innervate multiple end organs on one side or even bifurcate within the brain to innervate both labyrinths (Schwarz et al., 1981; Eden and Correia, 1982; Strutz, 1982; Dechesne et al., 1984; Highstein and Baker, 1986; Valli et al., 1986; Dickman and Correia, 1992; Fujino et al., 1993; Marco et al., 1993; Birinyi et al., 2001; Chi et al., 2007).

Does the EVS participate in the adaptive processes observed after exposures to HG? We addressed this question indirectly in this study by (1) inferring EVS action on the hair cell by comparing Smax observed in each experimental group (Table 1), (2) inferring EVS action on the afferent by comparing average IR at Smax (IR, in ips/cycle) observed in each experimental group (Table 1), and (3) pairing the MIR (in ips) with and without EVS stimulation in absence of acceleration in the same afferent in each experimental group (Table 2). A more direct measure to determine EVS role in neural adaptation was sought, but not found. For example, axons presumed to originate from efferent neurons were too infrequently encountered and their discharge had no clear variants to permit a meaningful comparison among subjects within or across groups, and the EVS action on afferent sensitivity to acceleration stimuli via its influence on hair cell receptor potential could not be normalized in any meaningful manner, mostly due to the variability in EVS efficacy and frequency of occurrence within each fish.

Efferent vestibular system functional role in nonmammalian vertebrates is likely linked to innate behaviors, such as attack and feeding and escape and aversives established for survival of the animal and its reproduction. In toadfish these actions are preceded by a stereotypic behavior, and it is powerfully evoked during the anticipatory phase of production of large, self-generated, and behaviorally relevant movements (Boyle and Highstein, 1984, 1990b; Highstein and Baker, 1985; Tricas and Highstein, 1990, 1991). EVS regulation of afferent sensitivity is likely through its action on hair cell modulation (Flock and Russell, 1976; Boyle et al., 2009; Rabbitt et al., 2010; Rohmann et al., 2015; Poppi et al., 2018). A novel finding supporting the importance of behavioral context in EVS function was recently reported by Chagnaud et al. (2015). In a semi-isolated preparation, which permits identifying the source of incoming signals, of larval *Xenopus* it was shown corollary discharge signals arising from the spinal central pattern generator cyclically modulated efferent neurons during fictive swimming. During these phasic epochs of efferent signaling, there was a corresponding gain reduction of afferent responses to vestibular-related stimuli. Thus, similar to toadfish, the EVS of *Xenopus* can effectively optimize sensory processing.

No presumed efferent neurons were encountered in the utricular nerve that consistently responded to applied otolith (linear acceleration or body roll/pitch) or canal (yaw rotation or head down/up pitch) stimuli. Given their low discharge at rest and disregard to benign vestibular stimulation, as well as the powerful excitation during preparatory behaviors, efferent neurons likely provide more episodic control over their synaptic targets and not a tonic influence. Recent evidence showed that the EVS could influence VOR plasticity using the α9 knockout mouse model, particularly after destruction of one labyrinth (Hübner et al., 2017). Although in a classical sense the EVS might still provide an underlying tonic drive to its targets, for the sake of argument in the present context a drive that also monitors the prevailing gravity condition, it does not provide cross-talk between otoliths and canals for continuous passive stimuli or influence the output during benign and nonthreatening volitional movements. Viewed from this perspective the EVS endows the toadfish with a powerful means to adjust the sensitivity and dynamic range of motion sensation, and is positioned to monitor prevailing conditions and regulate the gravity sensation (Kondrachuk and Boyle, 2006). However, no direct measurements or indirect inferences from afferent discharge behavior were found here to suggest a clear EVS role in regulating the utricular response to altered gravity. The information content provided by the efferent feedback signal in diverse conditions may be more complex and involve a novel self-regulation of synapses induced by changes in *input* signals in altered gravity.

## Author Contributions

All authors had full access to all the data in the study and take responsibility for the integrity of the data and the accuracy of the data analysis. RB research concept and design, and writing the article. RB, YP, and JV data acquisition, data analysis and interpretation, and statistical analysis.

## Conflict of Interest Statement

The authors declare that the research was conducted in the absence of any commercial or financial relationships that could be construed as a potential conflict of interest.

## References

[B1] Aceto J. Nourizadeh-Lillabadi R. Marée R. Dardenne N. Jeanray N. Wehenkel L. (2105). Zebrafish bone and general physiology are differently affected by hormones or changes in gravity. *PLoS One* 10:e0126928. 10.1371/journal.pone.0126928 26061167 PMC4465622

[B2] Angelaki D. E. (1991). Dynamic polarization vector of spatially tuned neurons. *IEEE Trans. Biomed. Eng.* 38 1053–1060. 10.1109/10.99068 1748439

[B3] Angelaki D. E. Bush G. A. Perachio A. A. (1993). Two-dimensional spatiotemporal coding of linear acceleration in vestibular nuclei neurons. *J. Neurosci.* 13 1403–1417. 8463828 10.1523/JNEUROSCI.13-04-01403.1993 PMC6576730

[B4] Anderson J. H. Blanks R. H. Precht W. (1978). Response characteristics of semicircular canal and otolith systems in cat. I. Dynamic responses of primary vestibular fibers. *Exp. Brain Res.* 32 491–507. 10.1007/BF00239549 28960

[B5] Angelaki D. E. Dickman J. D. (2000). Spatiotemporal processing of linear acceleration: primary afferent and central vestibular neuron responses. *J. Neurophysiol.* 84 2113–2132. 10.1152/jn.2000.84.4.2113 11024100

[B6] Anken R. H. (2006). On the role of the central nervous system in regulating the mineralisation of inner-ear otoliths of fish. *Protoplasma* 229 205–208. 10.1007/s00709-006-0219-6 17180502

[B7] Anken R. H. Beier M. Rahmann H. (2004). Hypergravity decreases carbonic anhydrase-reactivity in inner ear maculae of fish. *J. Exp. Zool.* 301A, 815–819. 10.1002/jez.a.97 15449341

[B8] Anken R. H. Rahmann H. (1998). Neurobiological responses of fish to altered gravity conditions: a review. *Acta Astronaut.* 42 431–454. 10.1016/S0094-5765(98)00137-4 11541626

[B9] Anken R. H. Werner K. Breuer J. Rahmann H. (2000a). Fish otolith growth in 1g and 3g depends on the gravity vector. *Adv. Space Res.* 25 2025–2029. 11542852 10.1016/s0273-1177(99)01010-8

[B10] Anken R. H. Edelmann E. Rahmann H. (2000b). Fish inner ear otoliths stop calcium incorporation after vestibular nerve transection. *Neuroreport* 11 2981–2983. 10.1097/00001756-200009110-00031 11006979

[B11] Aseyev N. Vinarskaya A. Roshchin M. Korshunova T. A. Malyshev A. Zuzina A. (2017). Adaptive changes in the vestibular system of land snail to a 30-day spaceflight and readaptation on return to Earth. *Front. Cell. Neurosci.* 11:348. 10.3389/fncel.2017.00348 29163058 PMC5672023

[B12] Baird R. A. Desmadryl G. Fernández C. Goldberg J. M. (1988). The vestibular nerve of the chinchilla. II. Relation between afferent response properties and peripheral innervation patterns in the semicircular canals. *J. Neurophysiol.* 60 182–203. 10.1152/jn.1988.60.1.182 3404216

[B13] Balaban P. M. Malyshev A. Y. Ierusalimsky V. N. Aseyev N. Korshunova T. A. Bravarenko N. I. (2011). Functional changes in the snail statocyst system elicited by microgravity. *PLoS One* 6:e17710. 10.1371/journal.pone.0017710 21479267 PMC3066201

[B14] Birinyi A. Straka H. Matesz C. Dieringer N. (2001). Location of dye-coupled second order and of efferent vestibular neurons labeled from individual semicircular canal or otolith organs in the frog. *Brain Res.* 921 44–59. 10.1016/S0006-8993(01)03075-X 11720710

[B15] Black F. O. Paloski W. H. Reschke M. F. Igarashi M. Guedry F. Anderson D. J. (1999). Disruption of postural readaptation by inertial stimuli following space flight. *J. Vestib. Res.* 9 369–378. 10544375

[B16] Boyle R. Carey J. P. Highstein S. M. (1991). Morphological correlates of response dynamics and efferent stimulation in horizontal semicircular canal afferents of the toadfish, *Opsanus tau*. *J. Neurophysiol.* 66 1504–1521. 10.1152/jn.1991.66.5.1504 1765791

[B17] Boyle R. Ehsanian R. Mofrad A. Popova Y. Varelas J. (2018). Morphology of the utricular otolith organ in the toadfish. *Opsanus tau*. *J. Comp. Neurol.* 2018 1–18. 10.1002/cne.24429 29524209 PMC5899691

[B18] Boyle R. Goldberg J. M. Highstein S. M. (1992). Inputs from regularly and irregularly discharging vestibular nerve afferents to secondary neurons in squirrel monkey vestibular nuclei. III. Correlation with vestibulospinal and vestibuloocular output pathways. *J. Neurophysiol.* 68 471–484. 10.1152/jn.1992.68.2.471 1527570

[B19] Boyle R. Highstein S. M. (1984). Effects of behavioral activation of the efferent vestibular system on the response dynamics of primary afferents of the horizontal semicircular canal in the toadfish. *Opsanus tau. Biol. Bull.* 167:523.

[B20] Boyle R. Highstein S. M. (1990a). Resting discharge and response dynamics of horizontal semicircular canal afferent of the toadfish. *Opsanus tau. J. Neurosci.* 10 1557–1569. 10.1523/JNEUROSCI.10-05-01557.1990 2332797 PMC6570080

[B21] Boyle R. Highstein S. M. (1990b). Efferent vestibular system in the toadfish: action upon horizontal semicircular canal afferents. *J. Neurosci.* 10 1570–1582. 2332798 10.1523/JNEUROSCI.10-05-01570.1990 PMC6570063

[B22] Boyle R. Mensinger A. F. Yoshida K. Usui S. Intravaia A. Tricas T. (2001). Neural readaptation to Earth’s gravity following return from space. *J. Neurophysiol.* 86 2118–2122. 10.1152/jn.2001.86.4.2118 11600668

[B23] Boyle R. Rabbitt R. D. Highstein S. M. (2009). Efferent control of hair cell and afferent responses in the semicircular canals. *J. Neurophysiol.* 102 1513–1525. 10.1152/jn.91367.2008 19571186 PMC2746798

[B24] Bracchi F. Gualierotti T. Morabito A. Rocca E. (1975). Multiday recordings from the primary neurons of the statoreceptors of the labyrinth of the bull frog. The effect of an extended period of “weightlessness” on the rate of firing at rest and in response to stimulation by brief periods of centrifugation (OFO-A orbiting experiment). *Acta Otolaryngol. Suppl.* 334 1–27. 1082707

[B25] Brichta A. M. Goldberg J. M. (2000). Responses to efferent activation and excitatory response-intensity relations of turtle posterior-crista afferents. *J. Neurophysiol.* 83 1224–1242. 10.1152/jn.2000.83.3.1224 10712451

[B26] Brown M. Nuttall A. Masta R. (1983). Intracellular recordings from cochlear inner hair cells: effects of stimulation of the crossed olivocochlear efferents. *Science* 222 69–72. 10.1126/science.6623058 6623058

[B27] Budelmann B. (1988). “Morphological diversity of equilibrium receptor systems in aquatic invertebrates,” in *Sensory Biology of Aquatic Animals*, eds Atema J. Fay R. Popper A. Tavolga W. (New York, NY: Springer), 757–782.

[B28] Chagnaud B. P. Banchi R. Simmers J. Straka H. (2015). Spinal corollary discharge modulates motion sensing during vertebrate locomotion. *Nat. Commun.* 4:7982. 10.1038/ncomms8982 26337184 PMC4569702

[B29] Chi F. L. Jiao Y. Liu H. J. Wang J. Shi Y. Barr J. J. (2007). Retrograde neuron tracing with microspheres reveals projection of CGRP-immunolabeled vestibular afferent neurons to the vestibular efferent nucleus in the brainstem of rats. *Neuroendocrinology* 85 131–138. 10.1159/000101959 17457027

[B30] Cohen B. Yakushin S. B. Holstein G. R. Dai M. Tomko D. L. Badakva A. M. (2005). Vestibular experiments in space. *Adv. Space Biol. Med.* 10 105–164. 10.1016/S1569-2574(05)10005-7 16101106 PMC12969068

[B31] Cotman C. W. Nieto-Sampedro M. (1982). Brain function, synapse renewal, and plasticity (1982). *Ann. Rev. Psychol.* 33 371–401. 10.1146/annurev.ps.33.020182.002103 6277238

[B32] Cragg B. G. (1969). Structural changes in naïve retinal synapses detectable within minutes of first exposure to daylight. *Brain Res.* 15 79–96. 10.1016/0006-8993(69)90311-4 5807783

[B33] Curthoys I. S. MacDougall H. G. Vidal P.-P. de Waele C. (2017). Sustained and transient vestibular systems: a physiological basis for interpreting vestibular function. *Front. Neurol.* 8:117. 10.3389/fneur.2017.00117 28424655 PMC5371610

[B34] Dechesne C. Raymond J. Sans A. (1984). The efferent vestibular system in the cat: a horseradish peroxidase and fluorescent retrograde tracers study. *Neuroscience* 11 893–901. 10.1016/0306-4522(84)90200-8 6204250

[B35] Demontis G. C. Germani M. M. Caiani E. G. Barravecchia I. Passino C. Angeloni D. (2017). Human pathophysiological adaptations to the space environment. *Front. Physiol.* 8:547 10.3389/fphys.2017.00547 PMC5539130 28824446

[B36] Desmond N. L. Levy W. B. (1986). Changes in the numerical density of synaptic contacts with long-term potentiation in the hippocampus dentate gyms. *J. Comp. Neurol.* 253 466–475. 10.1002/cne.902530404 3025272

[B37] Dickman J. D. Correia M. J. (1992). Vestibular efferent system in pigeons. Anatomical organization and effect upon semicircular canal afferent responsiveness. *Ann. N. Y. Acad. Sci.* 656 927–930. 10.1111/j.1749-6632.1992.tb25297.x 1599224

[B38] Eatock R. A. Corey D. P. Hudspeth A. J. (1987). Adaptation of mechanoelectrical transduction in hair cells of the bullfrog’s sacculus. *J. Neurosci.* 7 2821–2836. 10.1523/JNEUROSCI.07-09-02821.1987 3498016 PMC6569155

[B39] Eden A. R. Correia M. J. (1982). Identification of multiple groups of efferent vestibular neurons in the adult pigeon using horseradish peroxidase and DAPI. *Brain Res.* 248 201–208. 10.1016/0006-8993(82)90578-9 6982742

[B40] Fernández C. Goldberg J. M. (1971). Physiology of peripheral neurons innervating semicircular canals of the squirrel monkey. II. Response to sinusoidal stimulation and dynamics of peripheral vestibular system. *J. Neurophysiol.* 34 661–675. 10.1152/jn.1971.34.4.661 5000363

[B41] Fernández C. Goldberg J. M. (1976a). Physiology of peripheral neurons innervating otolith organs of the squirrel monkey. I. Response to static tilts and to long-duration centrifugal force. *J. Neurophysiol.* 39 970–984. 824412 10.1152/jn.1976.39.5.970

[B42] Fernández C. Goldberg J. M. (1976b). Physiology of peripheral neurons innervating otolith organs of the squirrel monkey. II. Directional selectivity and force-response relations. *J. Neurophysiol.* 39 985–995. 824413 10.1152/jn.1976.39.5.985

[B43] Fernández C. Goldberg J. M. (1976c). Physiology of peripheral neurons innervating otolith organs of the squirrel monkey. III. Response dynamics. *J. Neurophysiol.* 39 996–1008. 824414 10.1152/jn.1976.39.5.996

[B44] Flock A. Russell I. (1976). Inhibition by efferent nerve fibres: action on hair cells and afferent synaptic transmission in the lateral line canal organ of the burbot, lota lota. *J. Physiol.* 257 45–62. 10.1113/jphysiol.1976.sp011355 948076 PMC1309343

[B45] Fredrickson-Hemsing L. Strimbu C. E. Roongthumskul Y. Bozovic D. (2012). Dynamics of freely oscillating and coupled hair cell bundles under mechanical deflection. *Biophys. J.* 102 1785–1792. 10.1016/j.bpj.2012.03.017 22768934 PMC3328720

[B46] Friedman M. Giles S. (2017). “Actinopterygians: the ray-finned fishes – an explosion of diversity,” in *Evolution of the Vertebrate Ear: Evidence from the Fossil Record*, eds Clack J. Fay R. R. Popper A. N. (New York, NY: Springer).

[B47] Fujino K. Ito J. Tsuji J. (1993). Efferent vestibular fibers to otolith organs in guinea pigs. *Acta Otolaryngol.* 113 598–600. 10.3109/00016489309135870 8266785

[B48] Goldberg J. M. Desmadryl G. Baird R. A. Fernández C. (1990a). The vestibular nerve of the chinchilla. IV. Discharge properties of utricular afferents. *J. Neurophysiol.* 63 781–790. 2341876 10.1152/jn.1990.63.4.781

[B49] Goldberg J. M. Desmadryl G. Baird R. A. Fernández C. (1990b). The vestibular nerve of the chinchilla. V. Relation between afferent discharge properties and peripheral innervation patterns in the utricular macula. *J. Neurophysiol.* 63 791–804. 2341877 10.1152/jn.1990.63.4.791

[B50] Goldberg J. M. Fernández C. (1980). Efferent vestibular system in the squirrel monkey: anatomical location and influence on afferent activity. *J. Neurophysiol.* 43 986–1025. 10.1152/jn.1980.43.4.986 6767000

[B51] Goldberg J. M. Wilson V. J. Cullen K. E. Angelaki D. E. Broussard A. M. Büttner-Ennever J. (2012). *The Vestibular System: a Sixth Sense.* Oxford: Oxford University Press 10.1093/acprof:oso/9780195167085.001.0001

[B52] Graydon C. W. Manor U. Kindt K. S. (2017). In vivo ribbon mobility and turnover of ribeye at zebrafish hair cell synapses. *Sci. Rep.* 7:7467. 10.1038/s41598-017-07940-z 28785118 PMC5547071

[B53] Gribenski A. Caston J. (1976). Tonic influence of the efferent vestibular system on the spontaneous afferent activity from semicircular canals in the frog (*Rana esculenta* L.). *Exp. Brain Res.* 26 275–283. 10.1007/BF00234932 1086793

[B54] Gribenski A. Caston J. (1976). Tonic influence of the efferent vestibular system on the spontaneous afferent activity from semicircular canals in the frog (*Rana esculenta* L.). *Exp. Brain Res.* 26 275–283. 10.1007/BF00234932 1086793

[B55] Groff J. Liberman M. (2003). Modulation of cochlear afferent response by the lateral olivocochlear system: activation via electrical stimulation of in the inferior colliculus. *J. Neurophysiol.* 90 3178–3200. 10.1152/jn.00537.2003 14615429

[B56] Hallgren E. Kornilova L. Fransen E. Glukhikh D. Moore S. T. Clément G. (2016). Decreased otolith-mediated vestibular response in 25 astronauts induced by long-duration spaceflight. *J. Neurophysiol.* 115 3045–3051. 10.1152/jn.00065.2016 27009158 PMC4922620

[B57] Hartmann R. Klinke R. (1980). Efferent activity in the goldfish vestibular nerve and its influence on afferent activity. *Pflügers Arch.* 388 123–128. 10.1007/BF00584117 7192849

[B58] Heer M. Paloski W. H. (2006). Space motion sickness: incidence, etiology, and countermeasures. *Auton. Neurosci.* 129 77–79. PMID: 16935570 10.1016/j.autneu.2006.07.014 16935570

[B59] Highstein S. M. Baker R. (1985). Action of the efferent vestibular system on primary afferents in the toadfish, *Opsanus tau*. *J. Neurophysiol.* 54 370–384. 10.1152/jn.1985.54.2.370 4031993

[B60] Highstein S. M. Baker R. (1986). Organization of the efferent vestibular nuclei and nerves of the toadfish, *Opsanus tau*. *J. Comp. Neurol.* 243 309–325. 10.1002/cne.902430303 2869067

[B61] Highstein S. M. Goldberg J. M. Moschovakis A. K. Fernández C. (1987). Inputs from regularly and irregularly discharging vestibular nerve afferents to secondary neurons in the vestibular nuclei of the squirrel monkey. II Correlation with output pathways of secondary neurons. *J. Neurophysiol.* 58 719–738. 10.1152/jn.1987.58.4.719 2445938

[B62] Holstein G. R. Martinelli G. P. Boyle R. Rabbitt R. D. Highstein S. M. (2004). Ultrastructural observations of efferent terminals in the crista ampullaris of the toadfish, *Opsanus tau*. *Exp. Brain Res.* 157 128–136. 10.1007/s00221-004-1898-x 15318400

[B63] Holt J. C. Lysakowski A. Goldberg J. M. (2006). Mechanisms of efferent-mediated responses in the turtle posterior crista. *J. Neurosci.* 26 13180–13193. 10.1523/JNEUROSCI.3539-06.2006 17182768 PMC4157627

[B64] Honrubia V. Hoffman L. F. Sitko S. Schwartz I. R. (1989). Anatomic and physiologic correlates in bullfrog vestibular nerve. *J. Neurophysiol.* 61 688–701. 10.1152/jn.1989.61.4.688 2786056

[B65] Hübner P. P. Khan S. I. Migliaccio A. A. (2017). The mammalian efferent vestibular system plays a crucial role in vestibule-ocular reflex compensation after unilateral labyrinthectomy. *J. Neurophysiol.* 117 1553–1568. 10.1152/jn.01049.2015 28077670 PMC5376604

[B66] Hudspeth A. J. (2008). Making an effort to listen: mechanical amplification in the ear. *Neuron* 59 530–545. 10.1016/j.neuron.2008.07.012 18760690 PMC2724262

[B67] Hudspeth A. J. Corey D. P. (1977). Sensitivity, polarity, and conductance change in the response of vertebrate hair cells to controlled mechanical stimuli. *Proc. Natl. Acad. Sci. U.S.A.* 74 2407–2411. 10.1073/pnas.74.6.2407 329282 PMC432181

[B68] Jamali M. Sadeghi S. G. Cullen K. E. (2009). Response of vestibular nerve afferents innervating the utricle and saccule during passive and active translations. *J. Neurophysiol.* 101 141–149. 10.1152/jn.91066.2008 18971293 PMC3815216

[B69] Kondrachuk A. Boyle R. (2006). Feedback hypothesis and the effects of altered gravity on formation and function of gravireceptors of mollusks and fish. *Arch. Ital. Biol.* 144 75–87. 16642787

[B70] Kornilova L. N. Naumov I. A. Azarov K. A. Sagalovitch V. N. (2012). Gaze control and vestibular-cervical-ocular responses after prolonged exposure to microgravity. *Aviat. Space Environ. Med.* 83 1123–1134. 10.3357/ASEM.3106.2012 23316540

[B71] Landolt J. P. Correia M. J. (1980). Neurodynamic response analysis of anterior semicircular canal afferents in the pigeon. *J. Neurophysiol.* 43 1746–1770. 10.1152/jn.1980.43.6.1746 6251181

[B72] Liberman M. C. (1980). Efferent synapses in the inner hair cell area of the cat cochlea: an electron microscopic study of serial sections. *Hear. Res.* 3 189–204. 10.1016/0378-5955(80)90046-5 7440423

[B73] Lim D. J. Stith J. A. Stockwell C. W. Oyama J. (1974). Observations on saccules of rats exposed to long-term hypergravity. *Aerospace Med.* 45 705–710. 4837183

[B74] Loe P. R. Tomko D. L. Werner G. (1973). The neural signal of angular head position in primary afferent vestibular nerve axons. *J. Physiol.* 230 29–50. 10.1113/jphysiol.1973.sp010173 4702433 PMC1350384

[B75] Lysakowski A. Goldberg J. M. (2008). Ultrastructural analysis of the cristae ampullares in the squirrel monkey (*Saimiri sciureus*). *J. Comp. Neurol.* 511 47–64. 10.1002/cne.21827 18729176 PMC2828494

[B76] Marco J. Lee W. Suárez C. Hoffman L. Honrubia V. (1993). Morphologic and quantitative study of the efferent vestibular system in the chinchilla: 3-D reconstruction. *Acta Otolaryngol.* 113 229–234. 10.3109/00016489309135798 8517118

[B77] Marlinski V. Plotnik M. Goldberg J. M. (2004). Efferent actions in the chinchilla vestibular labyrinth. *J. Assoc. Res. Otolaryngol.* 5 126–143. 10.1007/s10162-003-4029-7 15357416 PMC2538405

[B78] Matsubara T. Uehara K. (2016). Homeostatic plasticity achieved by incorporation of random fluctuations and soft-bounded hebbian plasticity in excitatory synapses. *Front. Neural Circuits* 10:42. 10.3389/fncir.2016.00042 27313513 PMC4887490

[B79] Matthews G. Fuchs P. (2010). The diverse roles of ribbon synapses in sensory neurotransmission. *Nat. Rev. Neurosci.* 11 812–822. 10.1038/nrn2924 21045860 PMC3065184

[B80] McArthur K. L. Dickman J. D. (2008). Canal and otolith contributions to compensatory tilt responses in pigeons. *J. Neurophysiol.* 100 1488–1497. 10.1152/jn.90257.2008 18632885 PMC2544472

[B81] Moser T. Brandt A. Lysakowski A. (2006). Hair cell ribbon synapses. *Cell Tissue Res.* 326 347–359. 10.1007/s00441-006-0276-3 16944206 PMC4142044

[B82] Nilsson D.-E. (2013). Eye evolution and its functional basis. *Vis. Neurosci.* 30L, 5–20. 10.1017/S0952523813000035 23578808 PMC3632888

[B83] Oman C. M. (1998). Sensory conflict theory and space sickness: our changing perspective. *J. Vestib. Res.* 1 51–56. 10.1016/S0957-4271(97)00040-2 9416589

[B84] Paloski W. H. Black F. O. Reschke M. F. Calkins D. S. Shupert C. (1993). Vestibular ataxia following shuttle flights: effects of microgravity on otolith-mediated sensorimotor control of posture. *Am. J. Otol.* 14 9–17. 8424485

[B85] Pan B. Akyuz N. Liu X.-P. Asai Y. Nist-Lund C. Kurima K. (2018). TMC1 forms the pore of mechanosensory transduction channels in vertebrate inner ear hair cells. *Neuron* 99 736.e6–753.e6. 10.1016/j.neuron.2018.07.033 30138589 PMC6360533

[B86] Poppi L. A. Tabatabaee H. Drury H. R. Jobling P. Callister R. J. Migliaccio A. A. (2018). ACh-mediated hyperpolarization and decreased resistance in mammalian type II vestibular hair cells. *J. Neurophysiol.* 119 312–325. 10.1152/jn.00030.2017 28978760 PMC6048467

[B87] Puro D. G. De Mello F. G. Nirenberg M. (1977). Synapse turnover: the formation and termination of transient synapses. *Proc. Natl. Acad. Sci. U.S.A.* 74 4977–4981. 10.1073/pnas.74.11.4977 270733 PMC432081

[B88] Rabbitt R. D. Boyle R. Highstein S. M. (2010). Mechanical amplification by hair cells in the semicircular canals. *Proc. Natl. Acad. Sci. U.S.A.* 107 3864–3869. 10.1073/pnas.0906765107 20133682 PMC2840494

[B89] Rabbitt R. D. Brownell W. E. (2011). Efferent modulation of hair cell function. Curr. opin. otolaryngol. *Head Neck Surg.* 19 376–381. 10.1097/MOO.0b013e32834a5be1 22552698 PMC3343276

[B90] Reason J. T. Brandt J. J. (1975). *Motion Sickness.* London: Academic Press.

[B91] Regus-Leidig H. Tom Dieck S. Specht D. Meyer L. Brandstatter J. H. (2009). Early steps in the assembly of photoreceptor ribbon synapses in the mouse retina: the involvement of precursor spheres. *J. Comp. Neurol.* 512 814–824. 10.1002/cne.21915 19067356

[B92] Reschke M. F. Bloomberg J. J. Paloski W. H. Harm D. L. Parker D. E. (1994). “Neurophysiological aspects: sensory and sensory-motor function,” in *Space Physiology and Medicine*, ed. Pool S. L. (Philadelphia: Lea and Febiger), 261–285.

[B93] Rohmann K. N. Wersinger E. Braude J. P. Pyott S. J. Fuchs P. A. (2015). Activation of BK and SK channels by efferent synapses on outer hair cells in high-frequency regions of the rodent cochlea. *J. Neurosci.* 35 1821–1830. 10.1523/JNEUROSCI.2790-14.2015 25653344 PMC4315822

[B94] Ross M. D. (2000). Changes in ribbon synapses and rough endoplasmic reticulum of rat utricular macular hair cells in weightlessness. *Acta Otolaryngol.* 120 490–499. 10.1080/000164800750045983 10958400

[B95] Russell I. J. Murugasu E. (1997). Medial efferent inhibition suppresses basilar membrane responses to near characteristic frequency tones of moderate to high intensities. *J. Acoust. Soc. Am.* 102 1734–1738. 10.1121/1.420083 9301050

[B96] Sadeghi S. G. Goldberg J. M. Minor L. B. Cullen K. E. (2009). Efferent-mediated responses in vestibular nerve afferents of the alert macaque. *J. Neurophysiol.* 101 988–1001. 10.1152/jn.91112.2008 19091917 PMC2657077

[B97] Sans A. Highstein S. M. (1984). New ultrastructural features in the vestibular labyrinth of the toadfish. *Opsanus tau. Brain Res.* 308 191–195. 10.1016/0006-8993(84)90936-3 6332657

[B98] Schmidt F. Drenckhahn D. (1993). Intermediate stages in the disassembly of synaptic ribbons in cone development of the crucian carp, *Carassius-Carassius*. *Cell Tissue Res.* 272 487–490. 10.1007/BF00318554

[B99] Schneider A. D. Jamali M. Carriot J. Chacron M. J. Cullen K. E. (2015). The increased sensitivity of irregular peripheral canal and otolith vestibular afferents optimizes their encoding of natural stimuli. *J. Neurosci.* 35 5522–5536. 10.1523/JNEUROSCI.3841-14.2015 25855169 PMC4388918

[B100] Schwarz I. E. Schwarz D. W. Fredrickson J. M. Landolt J. P. (1981). Efferent vestibular neurons: a study employing retrograde tracer methods in the pigeon (*Columba livia*). *J. Comp. Neurol.* 196 1–12. 10.1002/cne.901960102 7204661

[B101] Sebastian C. Esseling K. Horn E. (2001). Altered gravitational forces affect the development of the static vestibuloocular reflex in fish (*Oreochromis mossambicus*). *J. Neurobiol.* 46 59–72. 10.1002/1097-4695(200101)46:1<59::AID-NEU6>3.0.CO;2-X 11108616

[B102] Shotwell S. L. Jacobs R. Hudspeth A. J. (1981). Directional sensitivity of individual vertebrate hair cells to controlled deflection of their hair bundles. *Ann. N. Y. Acad. Sci.* 374 1–10. 10.1111/j.1749-6632.1981.tb30854.x 6978627

[B103] Si X. Angelaki D. E. Dickman J. D. (1997). Response properties of pigeon otolith afferents to linear acceleration. *Exp. Brain Res.* 117 242–250. 10.1007/s002210050219 9419070

[B104] Smith C. A. Rasmussen G. L. (1968). “Nerve endings in the maculae and cristae of the chinchilla vestibule, with special reference to the efferents,” in *Proceedings of the 3rd Symposium role of the Vestibular Organs in Space Exploration*, (Washington, DC: NASA), 183–201.

[B105] Sondag H. N. de Jong H. A. Marle J. Oosterveld W. J. (1995). Effects of sustained acceleration on the morphological properties of otoconia in hamsters. *Acta Otolaryngol.* 115 227–230. 10.3109/00016489509139297 7610810

[B106] Spadero A. de Simone I. Puzzolo D. (1978). Ultrastructural data and chronobiological patterns of the synaptic ribbons in the outer plexiform layer in the retina of albino rats. *Acta Anat.* 102 365–373. 10.1159/000145659 696223

[B107] Spoendlin H. H. (1964). Organization of the sensory hairs in the gravity receptors in utricle and saccule of the squirrel monkey. *Z. Zellforsch.* 62 701–716. 10.1007/BF00341855 14219371

[B108] Spoon C. Moravec W. J. Rowe M. H. Grant J. W. Peterson E. H. (2011). Steady-state stiffness of utricular hair cells depends on macular location and hair bundle structure. *J. Neurophysiol.* 106 2950–2963. 10.1152/jn.00469.2011 21918003 PMC3234090

[B109] Stensiö E. A. (1927). The devonian and downtonian vertebrates of spitsbergen. 1. family cephalaspidae. *Skrifter om Svalbard og Ishavet* 12 1–391.

[B110] Straka H. Baker R. (2013). Vestibular blueprint in early vertebrates. *Front. Neural Circuits* 7:182. 10.3389/fncir.2013.00182 24312016 PMC3833255

[B111] Straka H. Fritzsch B. Glover J. C. (2014). Connecting ears to eye muscles: evolution of a ‘simple’ reflex arc. *Brain Behav. Evol.* 83 162–175. 10.1159/000357833 24776996

[B112] Strutz J. (1982). The origin of efferent vestibular fibres in the guinea pig. A horseradish peroxidase study. *Acta Otolaryngol.* 94 299–305. 10.3109/00016488209128917 7148442

[B113] Suli A. Pujol R. Cunningham D. E. Hailey D. W. Prendergast A. Rubel E. W. (2016). Innervation regulates synaptic ribbons in lateral line mechanosensory hair cells. *J. Cell Sci.* 129 2250–2260. 10.1242/jcs.182592 27103160 PMC4920245

[B114] Sultemeier D. R. Choy K. R. Schweizer F. E. Hoffman L. F. (2017). Spaceflight-induced synaptic modifications within hair cells of the mammalian utricle. *J. Neurophysiol.* 117 2163–2178. 10.1152/jn.00240.2016 28228581 PMC5454470

[B115] Tricas T. C. Highstein S. M. (1990). Visually mediated inhibition of lateral line primary afferent activity by the octavolateralis efferent system during predation in the free-swimming toadfish, *Opsanus tau*. *Exp. Brain Res.* 83 233–236. 10.1007/BF00232215 2073946

[B116] Tricas T. C. Highstein S. M. (1991). Action of the octavolateralis efferent system upon the lateral line of free-swimming toadfish, *Opsanus tau*. *J. Comp. Physiol.* 169 25–37. 10.1007/BF00198170 1941716

[B117] Valli P. Botta L. Zucca G. Casella C. (1986). Functional organization of the peripheral efferent vestibular system in the frog. *Brain Res.* 362 92–97. 10.1016/0006-8993(86)91402-2 3484653

[B118] Waites C. L. Craig A. M. Garner C. C. (2005). Mechanisms of vertebrate synaptogenesis. *Annu. Rev. Neurosci.* 28 251–274. 10.1146/annurev.neuro.27.070203.144336 16022596

[B119] Warr W. Guinan J. (1979). Efferent innervation of the organ of Corti: two separate systems. *Brain Res.* 173 152–155. 10.1016/0006-8993(79)91104-1 487078

[B120] Watt D. G. Money K. E. Tomi L. M. (1986). M.I.T./Canadian vestibular experiments on the Spacelab-1 mission: 3. Effects of prolonged weightlessness on a human otolith-spinal reflex. *Exp. Brain Res.* 64 308–315. 10.1007/BF00237748 3803475

[B121] Wersäll J. (1972). Morphology of the vestibular receptors in mammals. *Prog. Brain Res.* 37 3–17. 10.1016/S0079-6123(08)63890-X 4539320

[B122] Wiederhold M. L. Harrison J. L. Parker K. Nomura H. (2000). Otoliths developed in microgravity. *J. Grav. Physiol.* 7 39–42. 12697538

[B123] Wiederhold M. L. Pedrozo H. A. Harrison J. L. Hejl R. Gao W. (1997). Development of gravity-sensing organs in altered gravity conditions: opposite conclusions from an amphibian and a molluscan preparation. *J. Grav. Physiol.* 4 51–54. 11540698

[B124] Yu X.-J. Dickman J. D. Angelaki D. E. (2012). Detection thresholds of macaque otolith afferents. *J. Neurosci.* 32 8306–8316. 10.1523/JNEUROSCI.1067-12.2012 22699911 PMC3403680

[B125] Ziff E. B. Barry M. F. (2002). Receptor trafficking and the plasticity of excitatory synapses. *Curr. Opin. Neurobiol.* 12 279–286. 10.1016/S0959-4388(02)00329-X 12049934

