# Supplementary material for: Influence of Magnitude and Duration of Altered Gravity and Readaptation to 1 g on the Structure and Function of the Utricle in Toadfish, Opsanus tau
Source: Front Physiol. 2018 Oct 22;9:1469. doi: 10.3389/fphys.2018.01469 (PMC6204554; doi:10.3389/fphys.2018.01469)
Supplement: Supplementary file 1 [file Table_1.DOCX]

**Supplementary Material**

Influence of magnitude and duration of altered gravity and readaptation to 1g on the structure and function of the utricle in toadfish, *Opsanus tau*.

Richard Boyle*, Yekaterina Popova, and Joseph Varelas

*Correspondence to:

Richard Boyle

[richard.boyle@nasa.gov](mailto:richard.boyle@nasa.gov)

**Supplementary Figures**

**Supplementary Figure 1**

**
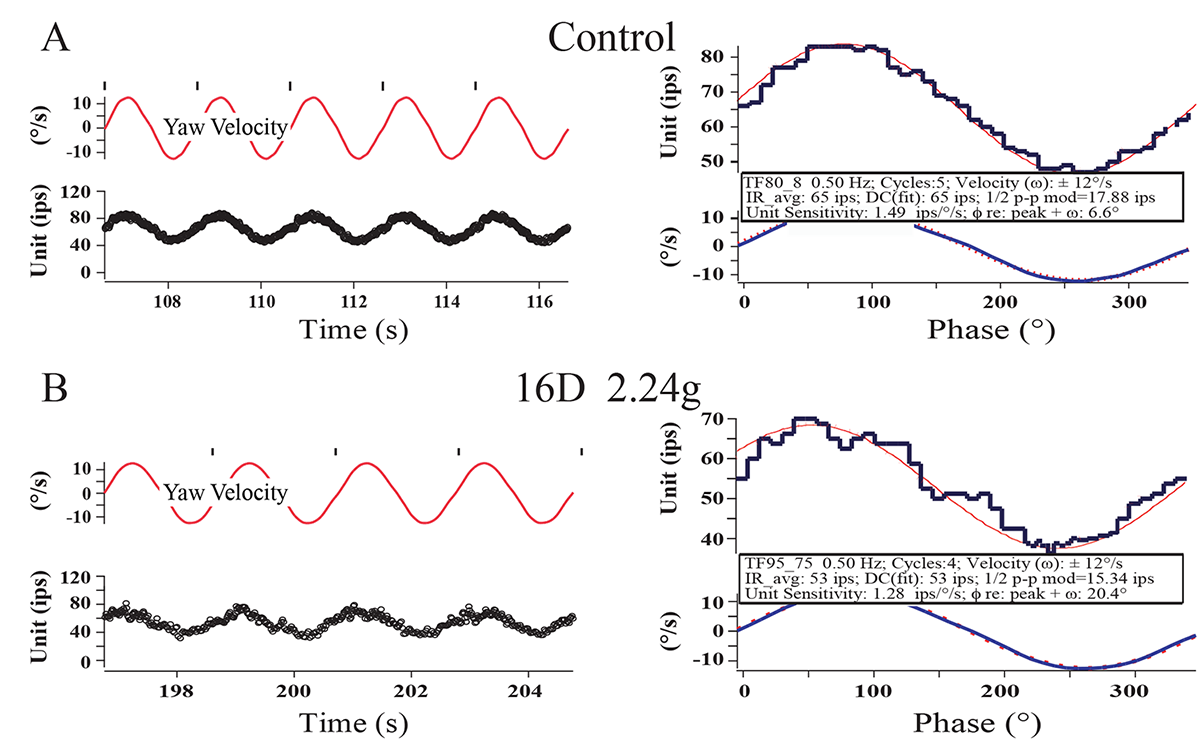
**

Normal horizontal semicircular canal response characteristics to yaw rotation after 2.24g centrifugation. As an internal control horizontal canal afferents were tested to EVS activation and sinusoidal yaw rotation from 0.2 – 5Hz (typically ±5 -30°/s) in every fish in every experimental group. (A) Left panel show IR of a canal afferent in a control fish to 5 continuous cycles of sinusoidal yaw rotation at 0.5Hz (±12°/s). Upper trace gives the yaw velocity (in ± °/s) with the tics at the zero-crossing to create the phase histogram of averaged response in right panel. Lower trace in left panel is instantaneous impulse rate. (B) Same format and stimulus parameters for a separate canal afferent recorded after 16 days of 2.24g. No detectable differences in IR or response to rotation were observed in the canal afferent population under these experimental conditions and a separate population of control afferents tested in an earlier study (Boyle and Highstein 1990a).

**Supplementary Figure 2**

**
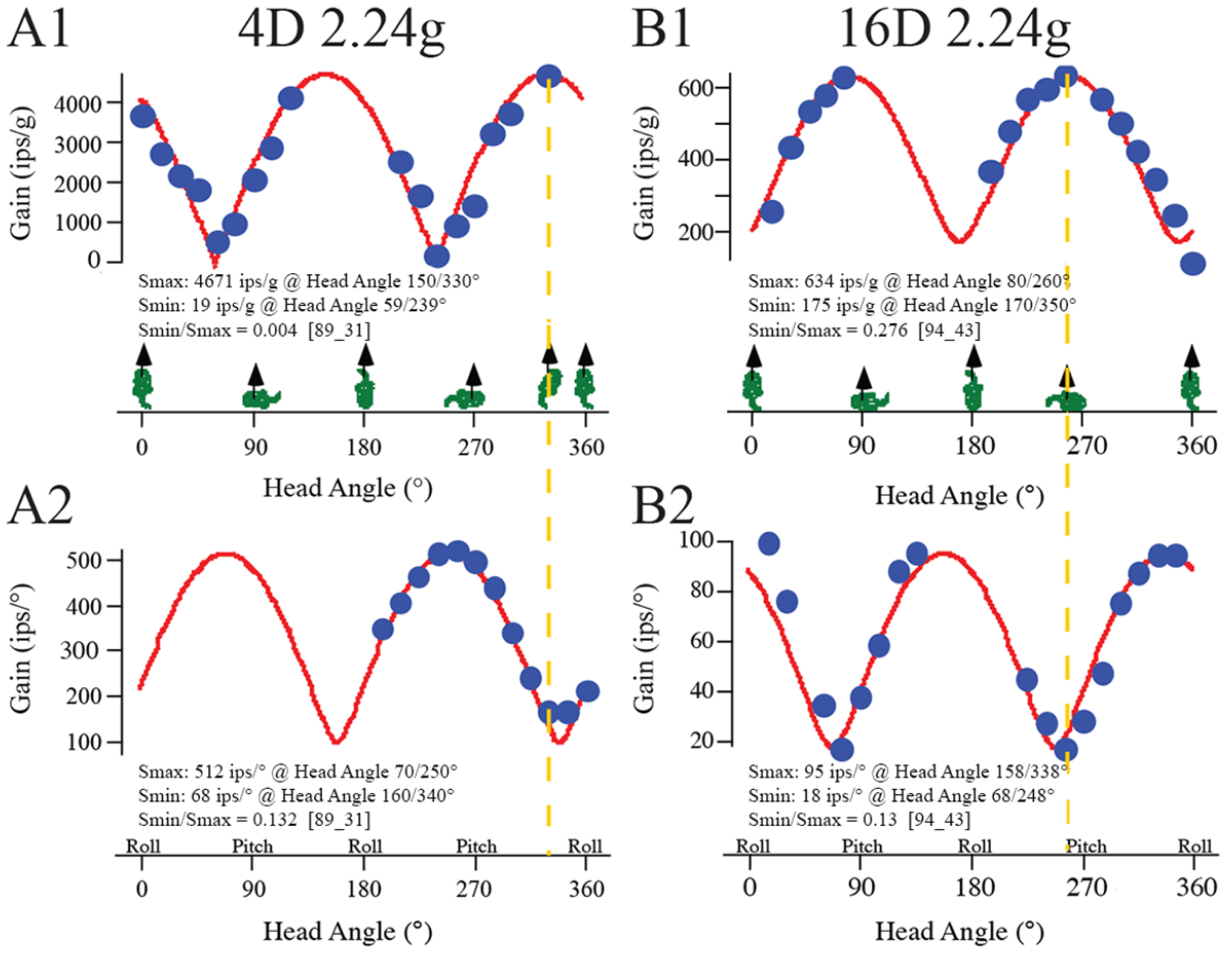
**

Utricular afferent responses to sinusoidal linear acceleration (upper panels, A1 and B1) and tilt (lower panels, A2 and B2) in fish exposed to 4 (A1, A2) or 16 (B1, B2) days at 2.24g. Same format as Fig. 2 (C,D). (A1, A2) Afferent recorded in a 4-Day centrifuged fish had a high Smax of 4671ips/g to linear acceleration and 512 ips/° to tilt, with a highly directional selectivity for linear acceleration (Smin/Smax = 0.004) and tilt (Smin/Smax = 0.132). (B1, B2) Afferent recorded in a 16-Day centrifuged fish had a low Smax of 634 ips/g to linear acceleration and 95 ips/° to tilt, with a directional selectivity for linear acceleration (Smin/Smax = 0.276) and tilt (Smin/Smax = 0.13).

**Supplementary Figure 3**


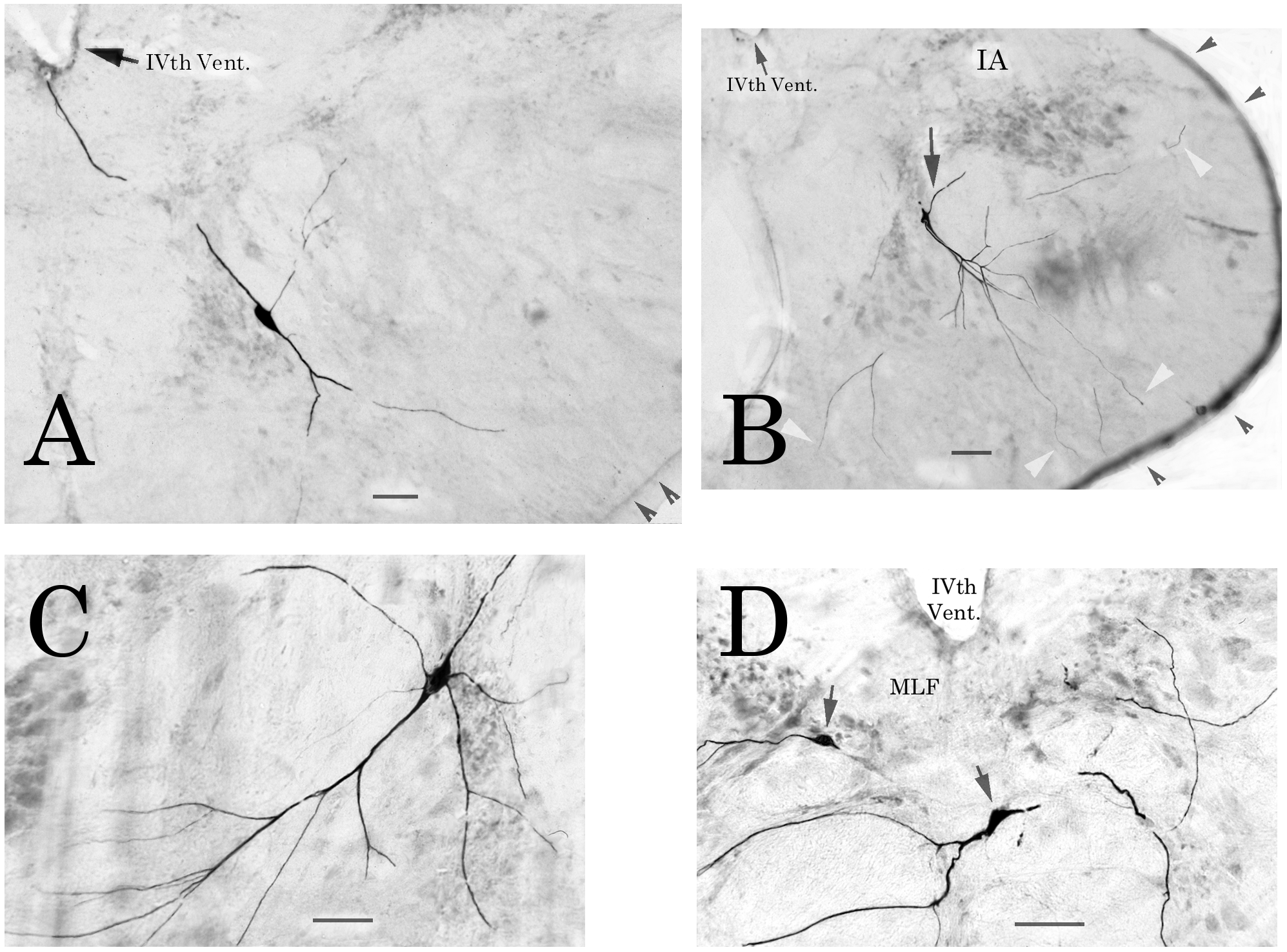


Brainstem morphology of efferent vestibular neurons innervating the utricle. Efferent neurons were retrogradely labeled by bulk application of biocytin into the partially severed, right utricular nerve. The utricular nerve was isolated from the nerves supplying the anterior and horizontal semicircular canals. The efferent neurons innervating the utricle are qualitatively similar to those described by Highstein and Baker (1985) using both intracellular injection of posterior canal efferent neurons and retrograde transport techniques from the nerves supplying the different endorgans with horseradish peroxidase. Utricular efferent neurons typically have extensive (A-D) dendritic trees are observed to extend bilaterally (A, D) and can span from the floor and dorsolateral border of the posterior medulla to the IVth ventricle (A, B; arrowheads). Two primary dendrites most commonly eminent from opposite poles of the cell body. When identified, the axon is seen leaving the cell body (B; arrow). A more elaborate dendritic structure is also seen (C). Labeled cell bodies were located both on the ipsi- and contralateral sides of the brainstem (D, left arrow). Abbreviations: IVth Vent., IVth Ventricle; MLF, medial longitudinal fasciculus; IA, internal arcuate. Calibration bars = 10 µm.
